# Changes in alcohol use during the COVID‐19 pandemic in Europe: A meta‐analysis of observational studies

**DOI:** 10.1111/dar.13446

**Published:** 2022-02-20

**Authors:** Carolin Kilian, Amy O'Donnell, Nina Potapova, Hugo López‐Pelayo, Bernd Schulte, Laia Miquel, Blanca Paniello Castillo, Christiane Sybille Schmidt, Antoni Gual, Jürgen Rehm, Jakob Manthey

**Affiliations:** ^1^ Institute of Clinical Psychology and Psychotherapy Technische Universität Dresden Dresden Germany; ^2^ Population Health Sciences Institute Newcastle University Newcastle upon Tyne UK; ^3^ Grup de Recerca en Addiccions Clinic Institut d'Investigacions Biomèdiques August Pi i Sunyer, Unitat Conductes Addictives Hospital Clínic Barcelona Spain; ^4^ Centre for Interdisciplinary Addiction Research University Medical Center Hamburg‐Eppendorf, Department of Psychiatry Hamburg Germany; ^5^ Department of Global Public Health (student) Karolinska Institute Stockholm Sweden; ^6^ Institute for Mental Health Policy Research Centre for Addiction and Mental Health Toronto Canada; ^7^ Dalla Lana School of Public Health University of Toronto Toronto Canada; ^8^ Faculty of Medicine, Institute of Medical Science University of Toronto, Medical Sciences Building Toronto Canada; ^9^ Campbell Family Mental Health Research Institute Centre for Addiction and Mental Health Toronto Canada; ^10^ Department of Psychiatry University of Toronto Toronto Canada; ^11^ I.M. Sechenov First Moscow State Medical University (Sechenov University) Moscow Russian Federation; ^12^ Department of Psychiatry, Medical Faculty University of Leipzig Leipzig Germany

**Keywords:** alcohol, drinking, COVID‐19, pandemic, Europe

## Abstract

**Issues:**

Numerous studies have examined the impact of the COVID‐19 pandemic on alcohol use changes in Europe, with concerns raised regarding increased use and related harms.

**Approach:**

We synthesised observational studies published between 1 January 2020 and 31 September 2021 on self‐reported changes in alcohol use associated with COVID‐19. Electronic databases were searched for studies evaluating individual data from European general and clinical populations. We identified 646 reports, of which 56 general population studies were suitable for random‐effects meta‐analyses of proportional differences in alcohol use changes. Variations by time, sub‐region and study quality were assessed in subsequent meta‐regressions. Additional 16 reports identified were summarised narratively.

**Key Findings:**

Compiling reports measuring changes in overall alcohol use, slightly more individuals indicated a decrease than an increase in their alcohol use during the pandemic [3.8%, 95% confidence interval (CI) 0.00–7.6%]. Decreases were also reported more often than increases in drinking frequency (8.0%, 95% CI 2.7–13.2%), quantity consumed (12.2%, 95% CI 8.3–16.2%) and heavy episodic drinking (17.7%, 95% CI 13.6–21.8%). Among people with pre‐existing high drinking levels/alcohol use disorder, high‐level drinking patterns appear to have solidified or intensified.

**Implications:**

Pandemic‐related changes in alcohol use may be associated with pre‐pandemic drinking levels. Increases among high‐risk alcohol users are concerning, suggesting a need for ongoing monitoring and support from relevant health‐care services.

**Conclusion:**

Our findings suggest that more people reduced their alcohol use in Europe than increased it since the onset of the pandemic. However high‐quality studies examining specific change mechanisms at the population level are lacking.

## Introduction

Major adverse events and crises affecting society can cause changes in alcohol use at the population level, as observed in the aftermath of economic crises [1], terrorist attacks [2] and natural disasters [3, 4]. Similarly, the spread of and responses to the severe acute respiratory coronavirus syndrome 2 (SARS‐CoV‐2; henceforth abbreviated as COVID‐19), declared a global pandemic by the World Health Organization (WHO) in March 2020 [5], may have resulted in shifts in alcohol use, with concerns expressed about possible increases in drinking levels in particular [6, 7, 8]. Alcohol is a major contributor to the burden of disease globally [9] and despite recent declines, Europe is the region with the highest per capita consumption worldwide, with three in five people consuming alcohol [10]. Monitoring pandemic‐related changes in alcohol use is therefore particularly important in this region.

Two major mechanisms have been hypothesised to influence changes in alcohol use during the pandemic. The first mechanism refers to increased levels of distress, both as a consequence of measures taken to contain the spread of the virus, such as social isolation, income insecurity and job loss [6, 11, 12, 13], as well as the threat of personal exposure to COVID‐19 or the illness of a loved one. Psychological pressure and distress are known risk factors for high levels of alcohol intake [14, 15] and therefore expected to lead to increased consumption during the COVID‐19 pandemic. In contrast, the second mechanism suggests a decline in alcohol use due to the reduced availability and affordability of alcoholic beverages during this period, as well as a reduction in potential drinking occasions due to measures aimed at limiting social gatherings [1, 6]. Evidence for this mechanism emerges from alcohol policy research, highlighting reduced availability and affordability of alcoholic beverages as effective policy measures to reduce alcohol use at the population level [16] (both policies are part of the WHO's ‘Best Buys’ to reduce alcohol use, see [17]). Both of these mechanisms may have played an important role in stimulating changes in alcohol use during the pandemic [18, 19, 20].

Preliminary findings from individual studies looking at alcohol use over the course of the pandemic suggest that changes in drinking differ by gender and age group [21, 22, 23, 24], and that there appears to have been a particular rise in alcohol intake in people reporting at‐risk drinking prior to March 2020 [18, 25]. Disparities in pandemic‐related changes in alcohol use across population groups have also been reported in narrative syntheses of published literature on changes in substance use during COVID‐19 [20, 26, 27, 28]. To date, however, there is a lack of robust quantitative assessments of the overall pattern of pandemic‐related changes in alcohol use across Europe. In response to this knowledge gap, we sought to review and meta‐analyse data from observational studies examining changes in alcohol use during the COVID‐19 pandemic in Europe. In doing so, we consider studies from general as well as from at‐risk populations, including people with alcohol use disorder (AUD), conduct gender‐stratified analyses and explore regional or time‐dependent patterns of consumption change over the pandemic period.

## Methods

### Search strategy and selection

A systematic literature search was conducted in Embase, MEDLINE and PubMed (via OVID) and the Web of Science on 1 October 2021 using appropriate search terms (see Table S1 for details). In addition, a Russian language literature search was conducted using CyberLeninka.ru and eLIBRARY.ru between 4 and 14 November 2021 using the same search terms. Eligible for inclusion were observational studies covering the general adult population or people with AUD that captured changes in alcohol use at any time since the COVID‐19 pandemic, were published since 1 January 2020 and located in Europe (using the UN Statistics Division definition [29] plus Cyprus, Georgia and Turkey). Studies focusing on aggregate or indirect measures of alcohol use, for example, alcohol purchases, were therefore excluded. English and Russian search terms were used to identify potentially eligible reports; however, searches were not restricted by language. Electronic database searches were supplemented with a grey literature search via Google Scholar and direct contact to colleagues in 47 countries to identify additional or non‐peer‐reviewed works not yet in the public domain.

Identified reports were screened in a two‐step process. First, titles and abstracts were screened by two independent reviewers against the pre‐specified inclusion criteria. Next, full texts of potentially eligible reports were similarly assessed by two additional independent reviewers. To ensure sufficient inter‐rater reliability [30], unclear decisions or disagreements at either step were discussed and resolved by the project lead. Studies identified in the grey literature search were screened separately by one reviewer and the project lead. The final list of included reports was again reviewed by experts in the field to ensure that no relevant studies were missed. The search strategy and review procedure were registered with PROSPERO (registration #: CRD42021238230) and complied with the PRISMA guidelines (see Table S2).

### Data extraction

The following information was extracted by one reviewer from included reports using a Microsoft Excel‐based data extraction template: study characteristics, including the study/survey name, the date of the study implementation, the country where the study was conducted and, if applicable, the region/city and the study design (cross‐sectional, repeated cross‐sectional or longitudinal study); sample size; the proportion of current drinkers; the proportion of women; mean age or age range; population subtype (general population, people with AUD) and subsample if available (i.e. results available for total sample only or stratified by gender); indicator of alcohol use change; outcome measure; and whether sampling weights were applied. We considered the following consumption change indicators: (i) changes in overall alcohol use; (ii) changes in the frequency of drinking; (iii) changes in the quantity of alcohol consumed on a usual drinking day; (iv) changes in heavy episodic drinking (HED) or binge drinking; and (v) for AUD populations only, risk of relapse. Outcome measures were either proportions or differences in the mean change in alcohol use during compared to prior the pandemic, as defined in the individual reports.

### Statistical analysis and data synthesis

Included reports were pooled by: (i) population subtype (general population, people with AUD); (ii) indicator of changes in alcohol use (overall, frequency, quantity, HED, relapse risk); and (iii) outcome measure (proportion change or mean change in alcohol use). Meta‐analyses were planned for all subsets where at least five independent studies were available [see 31]. A sufficient number of studies were identified for seven subsets of studies: (1) general population surveys of changes in overall alcohol use indicating the percentage of respondents who indicated to have decreased or increased their consumption, by women and men; general population surveys of changes in (1A) drinking frequency, (1B) quantity of alcohol consumed per drinking day, and (1C) frequency of HED; and (2) general population surveys of changes in the prevalence of alcohol use. The prevalence of alcohol use was defined as the proportion of subjects who either reported consuming alcohol at least once a week or once a month. We conducted a sensitivity analysis excluding reports based on convenience samples that measure changes in overall alcohol use in the total population.

Random‐effects meta‐analyses for the difference in: (i) the proportions of subjects reporting increases versus decreases; and (ii) the prevalence of alcohol users during versus before the pandemic were conducted using the function *rma* of the R package *metafor* [32]. Since the proportions refer to the same population, a difference for paired proportions was calculated by first calculating the difference between the number of respondents increasing their consumption (*n*_*inc*_) versus the number of respondents decreasing their consumption (*n*_*dec*_) and then dividing this difference by the number of alcohol users (*n*). Standard errors (*SE*) for each difference were used as source of variance and estimated based on the sample size of drinkers *n* and the number of respondents reporting decreases *n*_*dec*_ or increases *n*_*inc*_ (see Formula (1) [33]). To estimate the differences and standard errors in the prevalence of alcohol users, the same formulas were applied. Since not all studies reported the relevant information, sample sizes of alcohol users were estimated in some cases (*n* = 14 reports) by multiplying the total sample size (i.e. all participating respondents including people who do not drink alcohol) with the proportion of current drinkera and, in the case of gender‐stratified analyses, with the proportion of women/men. This procedure assumed that studies considered changes in alcohol use among current drinkers only. For studies not reporting the proportion of current drinkers, the total sample size was used. Very few studies also provided information on alcohol users starting or stopping drinking during the pandemic (*n* = 6). In these studies, the number of current drinkers and the number of those who stopped or started drinking were summed, and the corresponding proportions were accounted for as a decrease or increase in consumption, respectively, when reported individually. For one study providing monthly data on changes in alcohol use [34], proportions were averaged for each *a priori* defined pandemic period (March–June 2020, July–September 2020, October 2020 or later). Between‐study heterogeneity was evaluated using Cochran's *Q* and *I* ^2^ statistics. To identify possible publication bias, funnel plots were produced and inspected for symmetry, and statistically tested using Egger's regression‐based test [35]. To control for disproportional influence of any single study, leave‐one‐out analyses were performed.

(1) $$\mathrm{SE}=\frac{1}{n}\sqrt{n_{\mathrm{inc}}+n_{\mathrm{dec}}-\frac{{\left(n_{\mathrm{inc}}-n_{\mathrm{dec}}\right)}^{2}}{n}}$$

Finally, random‐effects meta‐regressions were repeated including possible moderators: timing of the study (categorical: March–June 2020, July–September 2020, October 2020 or later); a variable indicating an oversampling of younger or older adults (categorical: general population, at least half of the sample is younger or equal to 35 years, at least half of the sample is older or equal to 50 years); the inclusion of sampling weights (binary variable); and four European sub‐regions (categorial; see Table S3) [29]. Meta‐regressions were conducted separately for each moderator to proof for their independent impact, resulting in four analyses using the same set of studies. Therefore, a Bonferroni correction of the α = 0.05 significance threshold was applied, yielding a corrected *P*‐value of 0.0125 in moderator analyses [36]. All analyses were conducted in R version 4.1.1 [37] and the code is available upon request from the corresponding author.

For studies that did not qualify for meta‐analyses, brief narrative summaries of key findings are provided. This includes other studies from the general population whose outcomes were not comparable to those described above (e.g. prevalence of people reporting high‐risk drinking) as well as studies that looked at people with AUD. In the latter studies, we distinguished between findings relating to clinical population only, and findings that compared people with AUD with moderate drinkers.

Risk of bias for each study was assessed by one reviewer using an adapted version of the ROBINS‐I tool to meet the conditions for observational studies (available upon request) [38].

## Results

**Figure 1:**
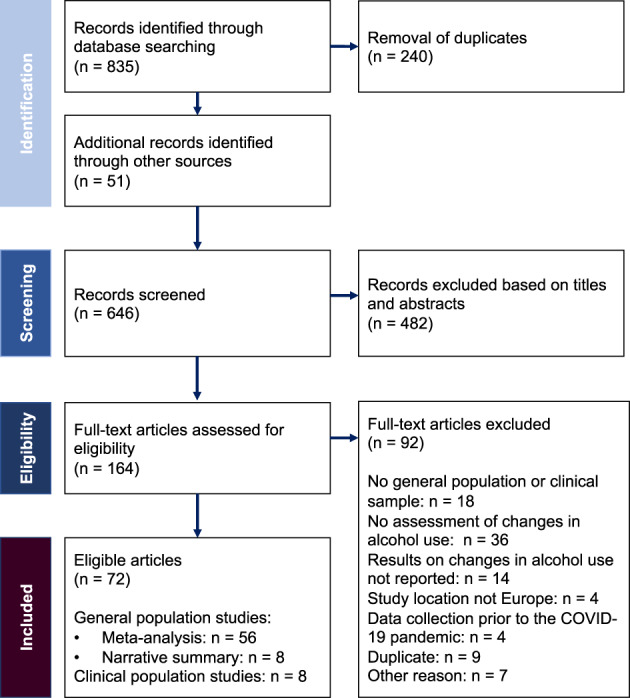
Flow chart on study selection.

We identified 646 reports of which 72 met our inclusion criteria (see Figure 1; one of which was retrieved from the search in the Russian language). Reasons for exclusion included, among others, a sample that did not reflect the general adult population or a clinical population, no assessment of changes in alcohol use or relevant results were not reported, a study location outside Europe and data collection prior to COVID‐19.

### Description of included reports

**Figure 2:**
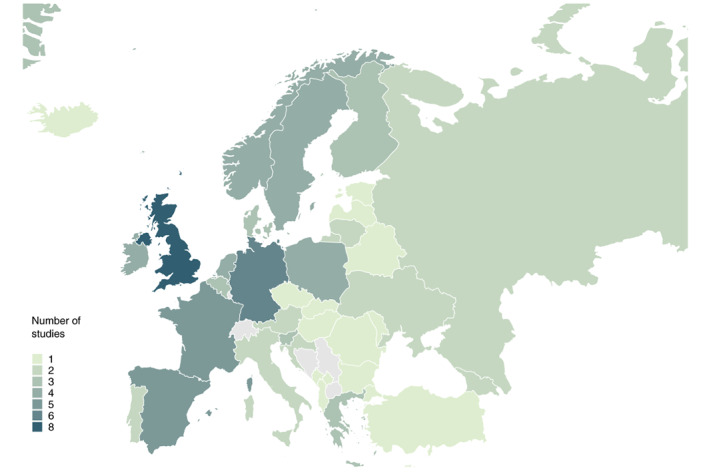
Countries covered in meta‐analysis and number of studies for each country. Countries not covered are grey (online version); Countries not covered are white (print version); Bosnia and Herzegovina as well as Serbia were included in a multi‐country study without country‐specific data being available [39].

The majority of reports comprised studies of the general adult population (*n* = 64), with only a small number of studies focussing on people with AUD (*n* = 8). Of those reports included in the meta‐analyses (*n* = 56), most covered individuals from the UK (*n* = 8), followed by Germany (*n* = 6), France (*n* = 5) and Spain (*n* = 5; see also Figure 2), and were carried out during the first months of the pandemic (March–June 2020, *n* = 44). Only a minority of these reports were repeated cross‐sectional (*n* = 3) or longitudinal studies (*n* = 1), while the majority constituted cross‐sectional surveys. Depending on the study design, changes in alcohol use were measured either as self‐perceived changes in alcohol use (e.g. “Has your consumption of alcohol changed?” [40]; *n* = 49), in pseudo pre‐post comparisons asking retrospectively about pre‐pandemic use (*n* = 6), or as true pre‐post assessments in longitudinal studies (*n* = 1). An overview of key characteristics of all studies included in the meta‐analyses is provided in Table S3. A brief overview of key findings of general population studies assessing changes in alcohol use which were not suitable for meta‐analyses is given in Table 1.

**Table 1:** Narrative summary of key findings of general population studies not included in meta‐analysis

Study	Country	Key study characteristics	Study period	Key findings
Cicero *et al*. [41]	Italy	Cross‐sectional survey study of a cohort of 359 elderly adults being at least 4 weeks in quarantine (strict lockdown), unweighted data	February–April 2020	Prevalence of respondents consuming more than one alcoholic drink per day increased from 1.4% to 25.6%. Overall, alcohol use significantly increased during quarantine as indicated by the share of alcohol in total energy intake (pre‐quarantine: 2.9% ± 0.6% versus during quarantine: 4.9% ± 1.0%).
Daly and Robinson [42]	UK	Longitudinal cohort study of 3358 middle‐aged adults, weighted data	May 2020 (compared to 2016–2018)	Significant increase in AUDIT‐PC score from 3.17 to 3.34 (*P* = 0.003) comparing 2016–2018 period to May 2020. Significant increases in high‐risk drinking (AUDIT‐PC scores ≥5) in women (14.0%–19.2%) and men (24.7% to 29.9%).
Laghi *et al*. [43]	Italy	Cross‐sectional survey study of 1533 young adults with women being over‐represented, unweighted data	April–May 2020	Mean AUDIT‐C score decreased for women from 2.33 (SD: 1.67) before the pandemic (retrospective assessment) to 1.51 (SD: 1.52) during the pandemic, and for young adult men from 3.00 (SD: 2.06) to 2.03 (SD: 1.88).
López‐Bueno *et al*. [44]	Spain	Cross‐sectional survey study of a convenience sample of adults being isolated in mandatory COVID‐19 confinement for at least 1 day (*n* = 2741), unweighted data	March–April 2020	Prevalence of any alcohol use decreased significantly with increasing length of COVID‐19 confinement, from 70.5% before COVID‐19 confinement to 53.4%, 46.5% and 43.4% at weeks 1, 2 and 3, respectively.
Marty *et al*. [45]	France	Cross‐sectional survey study of a convenience sample of 938 adults with women being overrepresented, unweighted data	April–May 2020	Prevalence of low and medium drinking levels (up to 100 g pure alcohol per week) increased significantly from 30% before to 39% during pandemic.
Skotnicka *et al*. 2021 [46]	Austria, Poland, UK	Cross‐sectional survey study including three convenience samples from Austria (*n* = 353), Poland (*n* = 407), and UK (*n* = 311), unweighted data	October 2020	Drinking alcohol at least weekly increased from before lockdown (retrospectively assessed) to the time of lockdown from 11.9% to 23.0% in Austria, from 16.2% to 23.1% in Poland and from 16.7% to 29.0% in the UK.
Studer *et al*. 2021 [47]	Switzerland	Longitudinal cohort study of 2344 young adult men, unweighted data	May–June 2020 (compared to April 2019 to early February 2020)	Weekly drinking volume and frequency of heavy episodic drinking significantly decreased during the pandemic compared to the pre‐pandemic period by 16.8% and 17.7%, respectively.
Villanueva *et al*. (2021) [48]	Spain	Cross‐sectional survey study of a convenience sample of 3779 adults, weighted data	April–May 2020	Medium‐ to high‐level drinking (AUDIT‐C ≥ 4 for women and ≥ 5 for men) significantly decreased from 16.1% pre‐pandemic to 9.3% during the pandemic.

AUDIT, Alcohol Use Disorders Identification Test.

### Risk of bias and methodological quality of studies

Quality assessment suggested that there was a serious (*n* = 46) or moderate (*n* = 11) risk of bias in most included reports. Only 15 studies were found to have a low risk of bias. The majority of studies either failed to properly report or had more than 20% missing values (*n* = 23), were found to be at serious risk of sampling bias by not weighting data derived from non‐probabilistic sampling techniques (*n* = 8), or both (*n* = 13; see Table S4). Given the very small number of studies with low or moderate risk of bias in the different meta‐analyses conducted (*n* < 5), no sensitivity analyses were performed excluding studies with serious risk of bias. Among reports included in meta‐analyses, almost three‐quarters did not apply statistical weights to their data regardless of applying a probabilistic or non‐probabilistic sampling approach (reports using weighted data: *n* = 25).

### Changes in overall alcohol use

**Figure 3:**
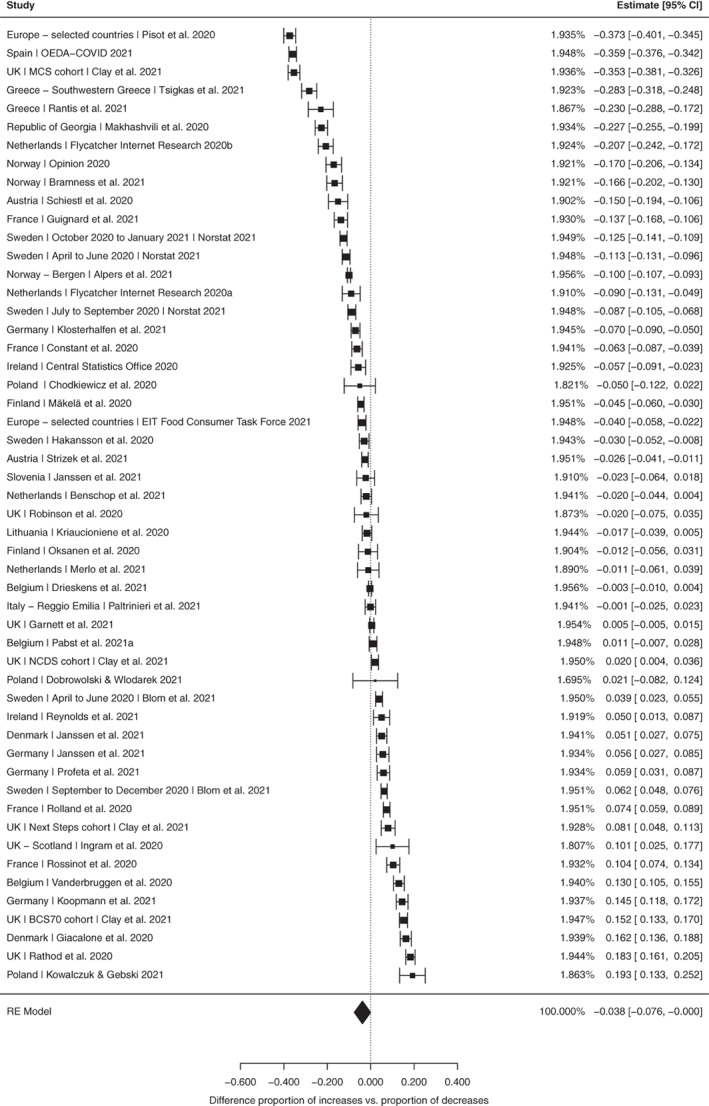
Random‐effects meta‐analysis for changes in alcohol use. Outcome measure was the difference in the proportion of respondents reporting increases minus decreases in alcohol use. Study details and references are provided in Table S4.

Forty‐four studies covering 189 321 current drinkers included information on proportional changes in overall alcohol use in either direction (i.e. decreases and increases). Among all studies, the pooled difference of the proportion of drinkers reporting increases minus the proportion of drinkers reporting decreases in overall alcohol use (referred to as ‘change score’ hereafter) was significant at −0.038 [95% confidence interval (CI) −0.076, 0.000; *P* = 0.048; see Figure 3]. This suggests that the group of drinkers indicating a reduction in their consumption during this period was slightly higher than the group of drinkers indicating an increase. Excluding reports based on convenience, samples resulted in a more pronounced difference between both groups (−0.075, 95% CI: −0.125, −0.024; *P* = 0.005; *n* = 21 studies; see Figure S1). Meta‐regression analyses showed no significant differences in the change scores by accounting for the moderator variables (see Table S5).

Pooling studies that reported results stratified by gender (women: 15 studies, *n* = 36 752; men: 13 studies, *n* = 26 376) found an overall effect that was not statistically different from zero for both women (−0.032, 95% CI −0.116, 0.051; *P* = 0.421) and men (−0.081, 95% CI −0.163, 0.001; *P* = 0.053; see Figures S2 and S3). Meta‐regression analyses revealed no significant differences in the change scores by any moderators in both subsamples (see Tables S6 and S7).

Between‐study heterogeneity was substantial in all four analyses (see Table 2), with *I* ^2^ ranging between 98.8% to 99.6% and Cochran's *Q* being significant (*P* < 0.001; for discussion, see Limitation section). There was no indication of publication bias (see Figures S4–S7) or of any study having a disproportional impact on the overall estimate based on leave‐one‐out analyses.

**Table 2:** Heterogeneity (Cochran's *Q* and *I* ^2^) and publication bias (Egger's regression‐based test and leave one out analysis) for main analysis

	Number of estimates	*Q* (*P*‐value)	*I* ^2^ (%)	Egger's t (*P*‐value)	Leave‐one‐out
*Changes in overall alcohol use*					
Total sample	52	6046.97 (<0.001)	99.4	0.131 (0.896)	No influence
Total sample—sensitivity analysis^a^	27	3928.72 (<0.001)	99.6	−1.429 (0.165)	No influence
Women	15	1319.73 (<0.001)	99.3	−0.294 (0.773)	No influence
Men	13	833.97 (<0.001)	98.8	−0.031 (0.976)	No influence
Changes in drinking frequency	41	1719.26 (<0.001)	98.4	−3.224 (0.003)	No influence
Changes in drinking quantity	39	905.29 (<0.001)	97.5	−1.812 (0.078)	No influence
Changes in the frequency of heavy episodic drinking	39	1610.43 (<0.001)	98.4	0.778 (0.442)	No influence
Change in prevalence of current alcohol consumers	10	44.98 (<0.001)	82.7	−0.364 (0.726)	Considerable influence of one study [49]

^a^ Reports based on general population samples were included only (*n* = 21).

### Changes in drinking frequency, quantity and heavy episodic drinking

Seven studies examined pandemic‐related changes in drinking frequency (*n* = 52 552) and six studies each on the quantity of alcohol consumed per drinking day (*n* = 47 318) and the frequency of HED (*n* = 51 940). We found a significant negative change score for drinking frequency, suggesting there was a larger proportion of respondents drinking less frequently than drinking more frequently during the pandemic (−0.080, 95% CI −0.132, −0.027; *P* = 0.004; see Figure S8). Moderator analyses revealed a varying change score by the timing of the assessment (see Table S8). In comparison to studies conducted between March and June 2020 (−0.026, 95% CI −0.091, 0.038; *P* = 0.412), those conducted in October 2020 or later had a significantly lower change score, indicating a significant decrease in drinking frequency during this later period (−0.163, 95% CI −0.245, −0.081; *P* < 0.001).

For changes in drinking quantity, a significant negative change score was observed (−0.122, 95% CI −0.162, −0.083; *P* < 0.001; see Figure S9), suggesting that the group of drinkers reporting decreases in the amount of alcohol consumed per drinking day were 12.2% points larger than those reporting increases. Meta‐regression analyses found no significant differences in the change scores by moderator variables (see Table S9).

Finally, a significant negative change score was identified for changes in the frequency of HED (−0.177, 95% CI −0.218, −0.136; *P* < 0.001; see Figure S10), indicating that a considerably higher proportion of drinkers decreased than increased occasions of HED. Meta‐regression analyses showed no significant differences in the change scores by moderator variables (see Table S10).

Between‐study heterogeneity was substantial in all three analyses, with *I* ^2^ ranging from 97.5% to 98.4% and Cochran's *Q* being significant (*P* < 0.001; see Table 2). For drinking frequency, but not for drinking quantity or the frequency of HED, a significant Egger's regression‐based test and asymmetric funnel plot indicated possible publication bias (see Figures S11–S13). Specifically, the asymmetric funnel plot suggests that studies with smaller standard errors (larger sample sizes) were more likely to report a positive change score, that is, an increase in drinking frequency (see Figure S11). In contrast, studies with larger standard errors (smaller sample sizes) were more likely to report a negative change score, that is, a decrease in drinking frequency. Leave‐one‐out analyses did not indicate that any study had a disproportionate influence on the overall estimates.

### Changes in the prevalence of alcohol use

The final meta‐analysis pooled seven studies (*n* = 25 697) that looked at the prevalence of alcohol intake during versus before the pandemic. The pooled difference was −0.076 (95% CI −0.119, −0.032; *P* = 0.003; see Figure S14), indicating that the prevalence of alcohol use during compared to before the pandemic has decreased by 6.9% points. Changes in prevalence did not vary across moderator variables (see Table S11). Between‐study heterogeneity was again substantial based on both indicators (see Table 2), while there was no indication of a publication bias (see Figure S15). Leave‐one‐out analysis identified one study whose exclusion led to a small decline in the pooled reduction of alcohol use prevalence (−0.064, 95% CI −0.103, −0.025; *P* = 0.006; *I* ^2^ = 74.4%; see Table 2).

### Narrative summary of alcohol use changes among people with AUD

An additional eight studies including people with AUD are summarised narratively hereafter (for key characteristics of these studies, see Table S12).

A cross‐sectional online survey of Polish adults assessed the impact of national lockdown (24 March–6 May 2020) on dietary habits, smoking and alcohol use [50]. The sample included a small number of respondents (*n* = 14, 1.2% of the sample) who described themselves as being alcohol dependent. While most respondents reported no increase in consumption during lockdown (77%), self‐declared dependent drinkers were more likely than other drinkers to report an increase during this period (64% vs. 14%). Comparable results were reported in a repeated cross‐sectional survey study from England [51]. High‐risk drinking was found to be increased by +39.5% during COVID‐19 compared to the pre‐pandemic period (August 2019–February 2020), with greater increases among women (+55.4%) compared to men (+30.7%), while a decrease of −7.8% was observed in the comparator year (April–July 2019 compared to August 2018–February 2019). In addition, there was a significant increase in alcohol reduction attempts during the pandemic compared to the pre‐pandemic period (+75.5%).

Another repeated cross‐sectional survey study of non‐institutionalised Czech adults examined the impact of the COVID‐19 pandemic on the prevalence of alcohol abuse and AUD [52]. Across three measurement points, the prevalence of AUD insignificantly fluctuated between 6.6% in November 2017, 4.3% in May 2020 and 5.0% in November 2020, and for alcohol abuse between 9.4%, 7.9% and 10.4%, respectively. Finally, a subsample of the Belgian online cross‐sectional survey study [53] were used to compare changes in respondents at high risk for AUD (*n* = 299, Alcohol Use Disorders Identification Test [AUDIT] score > 19) with non‐abstinent moderate drinkers (*n* = 299, matched controls, AUDIT scores 1–8) [54]. They found that although respondents at risk for AUD were more likely than moderate drinkers to modify their consumption following lockdown (91.3% vs. 71.6%, respectively), overall, they were actually more likely to decrease (65.9% vs. 35.1%, respectively) rather than increase their consumption (25.4% vs. 36.5%, respectively). At the same time, respondents with AUD at either end of the scale showed greater changes in consumption compared to moderate drinkers, that is, those who decreased consumption, decreased more and vice versa.

Studies focusing exclusively on people within the health‐care system stemmed from Spain (Barcelona, two studies), Russia (one study) and the UK (one study). One cross‐sectional online study assessed changes in the frequency of substance use among people seeking substance use disorder treatment, including AUD, in a specialised clinic in Barcelona [55]. Among all study participants, 18.9% reported a decrease, 12.5% reported an increase and 66% report no change in consumption compared to 6 months prior to lockdown. Similar findings were reported in a study based on patient reports of adults attending mental health services in Southern London [56]. While patients' mean AUDIT scores did not differ significantly before versus during the pandemic period, the prevalence of hazardous drinking decreased (17–8%), and the proportion of those potentially alcohol dependent increased (19–28%).

Only one study examined the impact of COVID‐19 on risk of relapse, analysing data from a retrospective cohort study of people with AUD (*n* = 362) attending an outpatient service at a specialist treatment hospital in Barcelona [57]. Results showed that the odds of patients screening positive for ethyl glucuronide (i.e. indicator for relapse) almost doubled during lockdown. Finally, a Russian study conducted in Moscow found that the number of people with severe alcohol poisoning increased fourfold between March and May 2020 compared to the same period of the previous year [58].

## Discussion

This systematic review and meta‐analysis synthesise the evidence from observational studies on changes in alcohol use during the COVID‐19 pandemic in Europe. Across 44 general population studies, the proportion of people reporting a decrease in overall alcohol use was slightly higher than the proportion of people reporting an increase over the course of the pandemic. In addition, a higher proportion of people reported drinking less frequently, consuming lower amounts of alcohol and having fewer HED occasions. The prevalence of alcohol use appears to have decreased during relative to the pre‐pandemic period. At the same time, evidence from the narrative summary suggests that, with few exceptions, high‐level drinking patterns solidified or even intensified during the pandemic among those with pre‐pandemic high drinking levels or AUD.

### Strengths and limitations

This study is among the first comprehensive efforts to synthesise research on alcohol use changes during the COVID‐19 pandemic in Europe. Key strengths of our review include the breadth of our search strategy, which enabled the identification of relevant peer‐reviewed and grey literature from across Europe. In addition, we employed a structured approach to data abstraction and used the recognised risk of bias tools to quality assess the literature. Finally, and importantly, unlike previous narrative summaries of this evidence, we used meta‐regression analyses where possible to quantify the size and direction of change in alcohol use during the pandemic. However, before we discuss our findings in a broader context, some limitations are outlined.

First, we found a serious risk of bias in a large number of included reports (see Table S4). Given the small number of studies with low or moderate risk of bias, a sensitivity analysis to exclude reports with a serious risk of bias was not possible. However, in our meta‐analysis, we did not find the consideration of sampling weights to affect pooled estimates. Second, most of the reports included based on convenience samples. The results of our sensitivity analysis, in which these reports were excluded, however, corroborated our main finding that more Europeans had reduced their alcohol use during the pandemic than increased it. This result is further consistent with figures of recorded alcohol *per capita* consumption as collected in the WHO monitoring efforts (yet unpublished analysis, personal communication of the head of the WHO Collaborating Centre collecting the data). Third, our analyses assumed that all changes in alcohol use reported in 2020 occurred in the context of the COVID‐19 pandemic. However, as most studies were of cross‐sectional design, no causal conclusions can be drawn, and secondary factors may also have had an impact on consumption changes. Fourth, our systematic literature search was conducted in English and Russian only, so relevant research published in other languages may not be covered. To ensure the best possible coverage of all relevant literature, we contacted researchers from more than 40 European countries as part of the grey literature search and asked them for national data on this topic.

Fifth, the interpretation of the meta‐analytical results is affected by considerable heterogeneity of the included reports and meta‐regressions could not identify an apparent single cause. Thus, a range of different factors may have contributed to the high level of heterogeneity. Previous research has questioned the ability of surveys to be representative of a country's population, particularly with regard to alcohol use [59, 60], with substantial underestimation of population‐level consumption [61, 62]. Furthermore, the validity of self‐report data on changes in alcohol use remains unclear. Apart from these methodological constraints, high heterogeneity could also be interpreted in light of the differential trends in consumption we identified. That is, rather than a simple one‐way effect (either a decrease or an increase in consumption), a shift in drinking appears to have taken place during the COVID‐19 pandemic, resulting in changes in consumption at both ends of the distribution. In other words, many people reduced their drinking, while at the same time some (particularly those with existing high drinking levels) increased their alcohol use [63]. Changes in consumption may thus be determined by pre‐pandemic drinking patterns; an indicator that could possibly explain some of the heterogeneity, but was barely considered in the reports included.

Finally, only a very limited number of studies provided information on people stopping or starting drinking alcohol during the COVID‐19 pandemic. In reports providing this information, more people reported to have stopped drinking alcohol during the pandemic (8.8–11.3%) than reported to have started alcohol use (0.3–6.3%) [64, 65, 66, 67].

### Interpretation

In line with recent research based on alcohol purchase data and other population consumption indicators, our findings could be interpreted as pointing towards a decrease in overall drinking levels in Europe during the COVID‐19 pandemic [68, 69, 70]. This decline, alongside the decreases we found in the prevalence of alcohol users, could be viewed as evidence to support the prior hypothesis, that is, that reduced availability and affordability of alcoholic beverages during the pandemic would result in a drop in alcohol use [6]. Other studies that examined the mechanism behind this change did not find conclusive evidence of reduced affordability of alcoholic beverages during 2020 [14, 19]. In Germany, for example, value‐added taxes were reduced in the second half of 2020, meaning alcoholic beverages actually became more affordable during the pandemic [68]. The extent to which reduced availability of alcohol due to the national measures adopted to curb the spread of COVID‐19 actually led to reduced consumption is also not yet clear. However, given the constraints on social gatherings and leisure activities commonly associated with elevated drinking, such as family celebrations, holidays, festivals and trips to bars and restaurants [71, 72, 73], it seems likely that social restrictions were an important driver behind the overall decrease in consumption [74]. In line with this, it is noteworthy that the most pronounced and consistent decline was observed in HED.

At the same time, while our meta‐analytical findings suggest that alcohol use declined during the pandemic, we found evidence of increasing alcohol use among those already drinking at high levels prior to COVID‐19. In line with the previously hypothesised distress mechanism [6, 11], coping with pandemic‐related stress is one of the most frequently mentioned motives for increased alcohol use [8, 14, 64, 74]. It is conceivable that individuals who were already drinking alcohol at high levels, including people with AUD, potentially consider drinking as a coping strategy and further increased their alcohol use during the pandemic in response to the numerous pandemic‐related stressors [7, 14]. Increased alcohol use among existing high‐level drinkers could have profound and negative public health consequences. There are already indications in some parts of Europe that the pandemic has exacerbated rates of alcohol‐related harm among this population. For example, one UK study found that primary care contacts decreased in the first half of 2020 for all health conditions examined, except for acute alcohol‐related events [75]. Furthermore, provisional mortality data from England and Wales show a marked increase in alcohol‐attributable deaths between April and September 2020, with a particularly rise in more deprived regions such as the North East of England [76]. Given that socio‐economically deprived communities already experienced disproportionate levels of alcohol‐related harm prior to COVID‐19 [77, 78], the pandemic has the potential to worsen health inequalities. Finally, not only may the pandemic change alcohol use, but alcohol can also adversely affect the infection with the coronavirus and the progression of COVID‐19 disease. This was shown in a large US case–control study, which found patients with AUD to have a significantly increased risk of COVID‐19 infection and worse outcomes compared to controls not having been diagnosed with a substance use disorder [79]. Others have highlighted the syndemic nature of COVID‐19, meaning that it interacts with and exacerbates existing social inequalities in chronic disease and the social determinants of health [80]. Research on the impact of COVID‐19 on alcohol‐related harm in Europe and other global regions is still at an early stage. Our findings highlight the need to rapidly expand the evidence base in this area, as well as to strengthen the public health response, particularly in the specialist treatment sector.

## Conflict of Interest

LM has received honoraria from GILEAD, Lundbeck and Neuraxpharm that are not related to this study. HLP has received training grants in the last 3 years from Lundbeck, Exeltis and Otsuka that are not related to this study.

## Supporting information


**Table S1:** Search terms.
**Table S2:** Prisma checklist (2020).
**Table S3:** Key characteristics of studies included in meta‐analysis.
**Table S4:** Risk of bias (ROB) assessment.
**Table S5:** Results for random‐effects meta‐regression analyses on the pooled change in overall alcohol use (total sample; 44 studies).
**Table S6:** Results for random‐effects meta‐regression analyses on the pooled change in overall alcohol use (women; 15 studies).
**Table S7:** Results for random‐effects meta‐regression analyses on the pooled change in overall alcohol use (men; 13 studies).
**Table S8:** Results for random‐effects meta‐regression analyses on the pooled change in drinking frequency (7 studies).
**Table S9:** Results for random‐effects meta‐regression analyses on the pooled change in the amount of alcohol consumed per drinking occasion (6 studies).
**Table S10:** Results for random‐effects meta‐regression analyses on the pooled change in the frequency of heavy episodic drinking (6 studies).
**Table S11:** Results for random‐effects meta‐regression analyses on the pooled change in the prevalence of alcohol users during versus before the pandemic (7 studies).
**Table S12:** Key characteristics of studies included in the narrative summary of alcohol use changes among people with alcohol use disorder.
**Figure S1:** Sensitivity analysis: random‐effects meta‐analysis for changes in alcohol use (n = 21); reports based on convenience samples were excluded. Outcome measure was the difference in the proportion of respondents reporting increases minus decreases in alcohol use.
**Figure S2:** Random‐effects meta‐analysis for changes in alcohol use among women. Outcome measure was the difference in the proportion of respondents reporting increases minus decreases in alcohol use.
**Figure S3:** Random‐effects meta‐analysis for changes in alcohol use among men. Outcome measure was the difference in the proportion of respondents reporting increases minus decreases in alcohol use.
**Figure S4:** Funnel plot for random‐effects meta‐analysis for changes in alcohol use for the total sample.
**Figure S5:** Funnel plot for sensitivity analysis.
**Figure S6:** Funnel plot for random‐effects meta‐analysis for changes in alcohol use for women.
**Figure S7:** Funnel plot for random‐effects meta‐analysis for changes in alcohol use for men.
**Figure S8:** Random‐effects meta‐analysis for changes in the drinking frequency. Outcome measure was the difference in the proportion of respondents reporting increases minus decreases in their drinking frequency.
**Figure S9:** Random‐effects meta‐analysis for changes in the drinking quantity. Outcome measure was the difference in the proportion of respondents reporting increases minus decreases in their drinking quantity.
**Figure S10:** Random‐effects meta‐analysis for changes in the frequency of heavy episodic drinking. Outcome measure was the difference in the proportion of respondents reporting increases minus decreases in their frequency of heavy episodic drinking.
**Figure S11:** Funnel plot for random‐effects meta‐analysis for changes in drinking frequency.
**Figure S12:** Funnel plot for random‐effects meta‐analysis for changes in drinking quantity.
**Figure S13:** Funnel plot for random‐effects meta‐analysis for changes in frequency of heavy episodic drinking.
**Figure S14:** Random‐effects meta‐analysis for changes in the prevalence of alcohol use. Outcome measure was the difference in the proportion of respondents reporting alcohol use during minus before the pandemic period.
**Figure S15:** Funnel plot for random‐effects meta‐analysis for changes in prevalence of alcohol use.
