# Supplementary material for: Changes in alcohol use during the COVID‐19 pandemic in Europe: A meta‐analysis of observational studies
Source: Drug Alcohol Rev. 2022 Feb 20;41(4):918–31. doi: 10.1111/dar.13446 (PMC9111882; doi:10.1111/dar.13446)
Supplement: Supplementary file 1 — Table S1: Search terms. Table S2: Prisma checklist (2020). Table S3: Key characteristics of studies included in meta‐analysis. Table S4: Risk of bias (ROB) assessment. Table S5: Results for random‐effects meta‐regression analyses on the pooled change in overall alcohol use (total sample; 44 studies). Table S6: Results for random‐effects meta‐regression analyses on the pooled change in overall alcohol use (women; 15 studies). Table S7: Results for random‐effects meta‐regression analyses on the pooled change in overall alcohol use (men; 13 studies). Table S8: Results for random‐effects meta‐regression analyses on the pooled change in drinking frequency (7 studies). Table S9: Results for random‐effects meta‐regression analyses on the pooled change in the amount of alcohol consumed per drinking occasion (6 studies). Table S10: Results for random‐effects meta‐regression analyses on the pooled change in the frequency of heavy episodic drinking (6 studies). Table S11: Results for random‐effects meta‐regression analyses on the pooled change in the prevalence of alcohol users during versus before the pandemic (7 studies). Table S12: Key characteristics of studies included in the narrative summary of alcohol use changes among people with alcohol use disorder. Figure S1: Sensitivity analysis: random‐effects meta‐analysis for changes in alcohol use (n = 21); reports based on convenience samples were excluded. Outcome measure was the difference in the proportion of respondents reporting increases minus decreases in alcohol use. Figure S2: Random‐effects meta‐analysis for changes in alcohol use among women. Outcome measure was the difference in the proportion of respondents reporting increases minus decreases in alcohol use. Figure S3: Random‐effects meta‐analysis for changes in alcohol use among men. Outcome measure was the difference in the proportion of respondents reporting increases minus decreases in alcohol use. Figure S4: Funnel plot for random‐effects meta‐analysis for c [file DAR-41-918-s001.docx]

# Supporting Information

**Title:** Changes in alcohol use during the COVID-19 pandemic in Europe: a meta-analysis of observational studies

**Running title:** Alcohol and COVID-19 in Europe: a meta-analysis

**Authors:** Carolin Kilian, Amy O’Donnell, Nina Potapova, Hugo López-Pelayo, Bernd Schulte, Laia Miquel, Blanca Paniello Castillo, Christiane Sybille Schmidt, Antoni Gual, Jürgen Rehm, Jakob Manthey

**Table of Contents**

[Table S1. Search terms. 3](#_Toc95473057)

[Table S2. Prisma checklist (2020). 5](#_Toc95473058)

[Table S3. Key characteristics of studies included in meta-analysis. 8](#_Toc95473059)

[Table S4. Risk of bias (ROB) assessment. 16](#_Toc95473060)

[Table S5. Results for random-effects meta-regression analyses on the pooled change in overall alcohol use (total sample; 44 studies). 18](#_Toc95473061)

[Table S6. Results for random-effects meta-regression analyses on the pooled change in overall alcohol use (women; 15 studies). 19](#_Toc95473062)

[Table S7. Results for random-effects meta-regression analyses on the pooled change in overall alcohol use (men; 13 studies). 20](#_Toc95473063)

[Table S8. Results for random-effects meta-regression analyses on the pooled change in drinking frequency (7 studies). 21](#_Toc95473064)

[Table S9. Results for random-effects meta-regression analyses on the pooled change in the amount of alcohol consumed per drinking occasion (6 studies). 22](#_Toc95473065)

[Table S10. Results for random-effects meta-regression analyses on the pooled change in the frequency of heavy episodic drinking (6 studies). 23](#_Toc95473066)

[Table S11. Results for random-effects meta-regression analyses on the pooled change in the prevalence of alcohol users during versus before the pandemic (7 studies). 24](#_Toc95473067)

[Table S12. Key characteristics of studies included in the narrative summary of alcohol use changes among people with alcohol use disorder 25](#_Toc95473068)

[Figure S1. Sensitivity analysis: random-effects meta-analysis for changes in alcohol use (n = 21); reports based on convenience samples were excluded. Outcome measure was the difference in the proportion of respondents reporting increases minus decreases in alcohol use. 26](#_Toc95473069)

[Figure S2. Random-effects meta-analysis for changes in alcohol use among women. Outcome measure was the difference in the proportion of respondents reporting increases minus decreases in alcohol use. 27](#_Toc95473070)

[Figure S3. Random-effects meta-analysis for changes in alcohol use among men. Outcome measure was the difference in the proportion of respondents reporting increases minus decreases in alcohol use. 28](#_Toc95473071)

[Figure S4. Funnel plot for random-effects meta-analysis for changes in alcohol use for the total sample. 29](#_Toc95473072)

[Figure S5. Funnel plot for sensitivity analysis. 30](#_Toc95473073)

[Figure S6. Funnel plot for random-effects meta-analysis for changes in alcohol use for women. 31](#_Toc95473074)

[Figure S7. Funnel plot for random-effects meta-analysis for changes in alcohol use for men. 32](#_Toc95473075)

[Figure S8. Random-effects meta-analysis for changes in the drinking frequency. Outcome measure was the difference in the proportion of respondents reporting increases minus decreases in their drinking frequency. 33](#_Toc95473076)

[Figure S9. Random-effects meta-analysis for changes in the drinking quantity. Outcome measure was the difference in the proportion of respondents reporting increases minus decreases in their drinking quantity. 34](#_Toc95473077)

[Figure S10. Random-effects meta-analysis for changes in the frequency of heavy episodic drinking. Outcome measure was the difference in the proportion of respondents reporting increases minus decreases in their frequency of heavy episodic drinking. 35](#_Toc95473078)

[Figure S11. Funnel plot for random-effects meta-analysis for changes in drinking frequency. 36](#_Toc95473079)

[Figure S12. Funnel plot for random-effects meta-analysis for changes in drinking quantity. 37](#_Toc95473080)

[Figure S13. Funnel plot for random-effects meta-analysis for changes in frequency of heavy episodic drinking. 38](#_Toc95473081)

[Figure S14. Random-effects meta-analysis for changes in the prevalence of alcohol use. Outcome measure was the difference in the proportion of respondents reporting alcohol use during minus before the pandemic period. 39](#_Toc95473082)

[Figure S15. Funnel plot for random-effects meta-analysis for changes in prevalence of alcohol use. 40](#_Toc95473083)

[References 41](#_Toc87997106)

**Table S1.** Search terms.

| **Web of Science** | TOPIC: (COVID* OR pandemic OR SARS-CoV-2 OR corona*)  **AND**  TOPIC: (alc* OR drinking OR (alc* AND (abuse OR misuse OR disorder OR depend* OR addict*))  **AND**  TOPIC: (Europ* OR Albania OR Andorra OR Armenia OR Austria OR Azerbaijan OR Belarus OR Belgium OR Bosnia OR Bulgaria OR Croatia OR Czech* OR Cyprus OR Denmark OR Estonia OR Finland OR France, OR Georgia OR Germany OR Greece OR Hungary OR Iceland OR Ireland OR Italy OR Kazakhstan OR Kosovo OR Latvia OR Liechtenstein OR Lithuania OR Luxembourg OR Macedonia OR Malta OR Moldova OR Monaco OR Montenegro OR Netherlands OR Norway OR Poland OR Portugal OR Macedonia OR Romania OR Russia* OR San Marino OR Serbia OR Slovakia OR Slovenia OR Spain OR Sweden OR Switzerland OR Turkey OR Ukraine OR United Kingdom OR Scotland OR England OR Wales)  Limit 1 to yr=”2020-Current” |
| --- | --- |
| **OVID SP** | (COVID$ OR pandemic OR SARS-CoV-2 OR corona$).ti,ab.  **AND**  (alc$ OR drinking OR (alc$ AND (abuse OR misuse OR disorder OR depend$ OR addict$)).ti,ab.  **AND**  (Europ$ OR Albania OR Andorra OR Austria OR Armenia OR Azerbaijan OR Belarus OR Belgium OR Bosnia OR Bulgaria OR Croatia OR Czech$ OR Cyprus OR Denmark OR Estonia OR Finland OR France, OR Georgia OR Germany OR Greece OR Hungary OR Iceland OR Ireland OR Italy OR Kazakhstan OR Kosovo OR Latvia OR Liechtenstein OR Lithuania OR Luxembourg OR Macedonia OR Malta OR Moldova OR Monaco OR Montenegro OR Netherlands OR Norway OR Poland OR Portugal OR Macedonia OR Romania OR Russia$ OR San Marino OR Serbia OR Slovakia OR Slovenia OR Spain OR Sweden OR Switzerland OR Turkey OR Ukraine OR United Kingdom OR Scotland OR England OR Wales).ti,ab.  Limit 1 to yr=”2020-Current” |

**Table S2.** Prisma checklist (2020).

| **Section and Topic** | **Item #** | **Checklist item** | **Location where item is reported** |
| --- | --- | --- | --- |
| **TITLE** | | |  |
| Title | 1 | Identify the report as a systematic review. | M1 |
| **ABSTRACT** | | |  |
| Abstract | 2 | See the PRISMA 2020 for Abstracts checklist. | M3 |
| **INTRODUCTION** | | |  |
| Rationale | 3 | Describe the rationale for the review in the context of existing knowledge. | M4 |
| Objectives | 4 | Provide an explicit statement of the objective(s) or question(s) the review addresses. | M4-M5 |
| **METHODS** | | |  |
| Eligibility criteria | 5 | Specify the inclusion and exclusion criteria for the review and how studies were grouped for the syntheses. | M5; Table S1 |
| Information sources | 6 | Specify all databases, registers, websites, organisations, reference lists and other sources searched or consulted to identify studies. Specify the date when each source was last searched or consulted. | M5 |
| Search strategy | 7 | Present the full search strategies for all databases, registers and websites, including any filters and limits used. | M5; Table S1 |
| Selection process | 8 | Specify the methods used to decide whether a study met the inclusion criteria of the review, including how many reviewers screened each record and each report retrieved, whether they worked independently, and if applicable, details of automation tools used in the process. | M5 |
| Data collection process | 9 | Specify the methods used to collect data from reports, including how many reviewers collected data from each report, whether they worked independently, any processes for obtaining or confirming data from study investigators, and if applicable, details of automation tools used in the process. | M5-M6 |
| Data items | 10a | List and define all outcomes for which data were sought. Specify whether all results that were compatible with each outcome domain in each study were sought (e.g. for all measures, time points, analyses), and if not, the methods used to decide which results to collect. | M5-M6 |
|  | 10b | List and define all other variables for which data were sought (e.g. participant and intervention characteristics, funding sources). Describe any assumptions made about any missing or unclear information. | M5-M6 |
| Study risk of bias assessment | 11 | Specify the methods used to assess risk of bias in the included studies, including details of the tool(s) used, how many reviewers assessed each study and whether they worked independently, and if applicable, details of automation tools used in the process. | M7 |
| Effect measures | 12 | Specify for each outcome the effect measure(s) (e.g. risk ratio, mean difference) used in the synthesis or presentation of results. | M6-M7 |
| Synthesis methods | 13a | Describe the processes used to decide which studies were eligible for each synthesis (e.g. tabulating the study intervention characteristics and comparing against the planned groups for each synthesis (item #5)). | M5-M6 |
|  | 13b | Describe any methods required to prepare the data for presentation or synthesis, such as handling of missing summary statistics, or data conversions. | M6-M7 |
|  | 13c | Describe any methods used to tabulate or visually display results of individual studies and syntheses. | M6-M7 |
|  | 13d | Describe any methods used to synthesize results and provide a rationale for the choice(s). If meta-analysis was performed, describe the model(s), method(s) to identify the presence and extent of statistical heterogeneity, and software package(s) used. | M6-M7 |
|  | 13e | Describe any methods used to explore possible causes of heterogeneity among study results (e.g. subgroup analysis, meta-regression). | M7 |
|  | 13f | Describe any sensitivity analyses conducted to assess robustness of the synthesized results. | M6-M7 |
| Reporting bias assessment | 14 | Describe any methods used to assess risk of bias due to missing results in a synthesis (arising from reporting biases). | M6-M7 |
| Certainty assessment | 15 | Describe any methods used to assess certainty (or confidence) in the body of evidence for an outcome. | M7 |
| **RESULTS** | | |  |
| Study selection | 16a | Describe the results of the search and selection process, from the number of records identified in the search to the number of studies included in the review, ideally using a flow diagram. | M8; Figure 1 |
|  | 16b | Cite studies that might appear to meet the inclusion criteria, but which were excluded, and explain why they were excluded. | Figure 1 |
| Study characteristics | 17 | Cite each included study and present its characteristics. | M8 (summary), Table S4, Table S12, Table 2 |
| Risk of bias in studies | 18 | Present assessments of risk of bias for each included study. | M8 (summary), Table S3 |
| Results of individual studies | 19 | For all outcomes, present, for each study: (a) summary statistics for each group (where appropriate) and (b) an effect estimate and its precision (e.g. confidence/credible interval), ideally using structured tables or plots. | Figure 3, Figures S1-S3, S8-S10, S14 |
| Results of syntheses | 20a | For each synthesis, briefly summarise the characteristics and risk of bias among contributing studies. | Table S3, Table S4, Table S12, Table 2 |
|  | 20b | Present results of all statistical syntheses conducted. If meta-analysis was done, present for each the summary estimate and its precision (e.g. confidence/credible interval) and measures of statistical heterogeneity. If comparing groups, describe the direction of the effect. | M9-M11, Figures S1-S3, S8-S10, S14; Table 2 |
|  | 20c | Present results of all investigations of possible causes of heterogeneity among study results. | Table 2 |
|  | 20d | Present results of all sensitivity analyses conducted to assess the robustness of the synthesized results. | M9; Tables S1, S8-S11 |
| Reporting biases | 21 | Present assessments of risk of bias due to missing results (arising from reporting biases) for each synthesis assessed. | Table S3 |
| Certainty of evidence | 22 | Present assessments of certainty (or confidence) in the body of evidence for each outcome assessed. | Figure 3, Figures S1-S3, S8-S^0, S14; Tables S5-S11 |
| **DISCUSSION** | | |  |
| Discussion | 23a | Provide a general interpretation of the results in the context of other evidence. | M12-15 |
|  | 23b | Discuss any limitations of the evidence included in the review. | M13-M14 |
|  | 23c | Discuss any limitations of the review processes used. | M13-M14 |
|  | 23d | Discuss implications of the results for practice, policy, and future research. | M14-M15 |
| **OTHER INFORMATION** | | |  |
| Registration and protocol | 24a | Provide registration information for the review, including register name and registration number, or state that the review was not registered. | M5 |
|  | 24b | Indicate where the review protocol can be accessed, or state that a protocol was not prepared. | M5 |
|  | 24c | Describe and explain any amendments to information provided at registration or in the protocol. | n. a. |
| Support | 25 | Describe sources of financial or non-financial support for the review, and the role of the funders or sponsors in the review. | M2 |
| Competing interests | 26 | Declare any competing interests of review authors. | M2 |
| Availability of data, code and other materials | 27 | Report which of the following are publicly available and where they can be found: template data collection forms; data extracted from included studies; data used for all analyses; analytic code; any other materials used in the review. | M7 |

M, manuscript, S, supplement

**Table S3.** Key characteristics of studies included in meta-analysis.

| **Study** | **Country** | **Study design** | **Study period** | **Sample size^1^** | **Sample characteristics** | **Change measure^2^** | |
| --- | --- | --- | --- | --- | --- | --- | --- |
| **Western European countries** | | | | | |  | |
| Schiestl 2020 – Austrian Corona Panel Project [1] | Austria | Cross-sectional survey study | April 2020 | 1527 (current alcohol users: 946) | General adult population, weighted data | 1 | |
| Strizek *et al*. 2021 [2] | Austria | Repeated cross-sectional survey study | First assessment: April to June, second assessment: October to November 2020 | 3289 (current alcohol users: 2605) | General adult population with oversampling of younger adults, weighted data | 1 | |
| Drieskens *et al*. 2021 [3] | Belgium | Cross-sectional survey study | April 2020 | 28,029 | General adult population sample, weighted sample | 1 | |
| Pabst *et al*. 2021a [4] | Belgium | Cross-sectional survey study | March to May 2020 | 7711 | Convenience sample of general adult population | 1 |  |
| Vanderbruggen *et al*. 2020 [5] | Belgium | Cross-sectional survey study | April 2020 | 3632 (current alcohol users: 2113) | Convenience sample of the general adult population, unweighted data | 1 | |
| Guignard *et al*. 2021 [6] | France | Cross-sectional survey study | March to April 2020 | 2003 (current alcohol users: 1344) | General adult population sample, weighted data | 1 | |
| Rolland *et al*. 2020 [7] | France | Cross-sectional survey study | March 2020 | 11,391 (current alcohol users: 7107) | General adult population sample, weighted sample | 1 | |
| Rossinot *et al*. 2020 [8] | France | Cross-sectional survey study | April to May 2020 | 1454 | Convenience sample of the general adult population, weighted data | 1 | |
| Constant *et al*. 2020 [9] | France | Cross-sectional survey study | April 2020 | 4005 (current alcohol users: 2409) | Convenience sample of the general adult population, unweighted data | 1 | |
| Klosterhalfen *et al*. 2021 [10] | Germany | Cross-sectional survey study | June to August 2020 | 4078 (current alcohol users: 2982) | Convenience sample of the general adult population, weighted data | 1 | |
| Koopmann *et al*. 2021 [11] | Germany | Cross-sectional survey study | April to May 2020 | 3245 (current alcohol users: 2962) | Convenience sample of the general adult population, women being overrepresented, unweighted data | 1 | |
| Profeta *et al*. 2021 [12] | Germany | Cross-sectional survey study | April 2020 | 973 | Convenience sample of the general adult population, unweighted data | 1 | |
| Schecke *et al*. 2021 [13] | Germany | Cross-sectional survey study | October to December 2020 | 2813 women | Convenience sample, unweighted data | 1 | |
| Benschop *et al*. 2021 [14] | Netherlands | Cross-sectional survey study | Mai to October 2020 | 6070 | Convenience sample of mostly young adult population (16+), unweighted data | 1, 2 | |
| Flycather Internet Research 2020a [15] | Netherlands | Cross-sectional survey study | April 2020 | April 2020: 1030 (current alcohol users: 683) | General adult population samples, unweighted data | 1 | |
| Flycather Internet Research 2020b [15] | Netherlands | Cross-sectional survey study | November 2020 | November 2020: 1265 (current alcohol users: 875) | General adult population samples, unweighted data | 1 | |
| Merlo *et al*. 2021 [16] | Netherlands | Cross-sectional survey study | June to July 2020 | 761 | Convenience sample of the general adult population, women being overrepresented, unweighted data | 1 | |
| **Northern European countries** | | | | | |  | |
| Giacalone *et al*. 2020 [17] | Denmark | Cross-sectional survey study | April to May 2020 | 2462 | Convenience sample of the general adult population, unweighted data | 1 | |
| Mäkelä *et al*. 2020 [18] | Finland | Cross-sectional survey study | April to June 2020 | 3525 | Convenience sample of the general adult population, weighted data | 1 | |
| Oksanen *et al*. 2021 [19] | Finland | Repeated cross-sectional survey study | March to April 2020 (compared to September to October 2019) | 1041 | General adult population sample, weighted sample | 1 | |
| Carbia *et al*. 2021 [20] | Ireland | Cross-sectional survey study | October to December 2020 | 773 (current alcohol users: 713) | Convenience sample of the general adult population, women being overrepresented, unweighted data | 1A-1C | |
| Central Statistics Office 2020 [21] | Ireland | Cross-sectional survey study | November 2020 | 1585 | General adult population sample, weighted data | 1 | |
| Reynolds *et al*. 2020 [22] | Ireland | Cross-sectional survey study | April 2020 | 1362 (current alcohol users: 1098) | General adult population sample, weighted data | 1 | |
| Kriaucioniene *et al*. 2020 [23] | Lithuania | Cross-sectional survey study | April 2020 | 2447 | Convenience sample of the general adult population, weighted data | 1 | |
| Bramness *et al*. 2020 [24] | Norway | Cross-sectional survey study | June to July 2020 | 1328 (current alcohol users: 1200) | General adult population sample, unweighted data | 1 | |
| Opinion 2020 [25] | Norway | Cross-sectional survey study | June to July 2020 | 1328 (current alcohol users: 1195) | General adult population sample, unweighted data | 1 | |
| Alpers *et al*. 2020 [26] | Norway (Bergen) | Cross-sectional survey study | April 2020 | 25,708 | General adult population sample, weighted data | 1 | |
| Blom *et al*. 2020 [27] | Sweden | Cross-sectional survey study | April to June, and September to December 2020 | 5599 | General adult population sample, unweighted data | 1 | |
| Håkansson *et al*. 2020 [28] | Sweden | Cross-sectional survey study | April to May 2020 | 2016 (current alcohol users: 1681) | General adult population sample, weighted data | 1 | |
| Norstat 2020 [29] | Sweden | Repeated cross-sectional survey study | Repeated measurement between April 2020 to January 2021 (compared to 2019) | 1000 per assessment | General adult population sample, unweighted data | 1 | |
| Clay *et al*. 2020 [30] | UK | Cross-sectional survey study | May 2020 | Four cohorts: 1852 to 4960 respondents | Four general population Millennium cohort (age 19), Millennial generation (age 30), 1970 British Cohort (age 50), and Baby Boomer generation (age 70) | 1 | |
| Garnett *et al*. 2021 [31] | UK | Cross-sectional survey study | March to April 2020 | 33,644 (current alcohol users: 21,212) | General adult population sample, weighted sample | 1 | |
| Miller *et al*. 2021 [32] | UK | Cross-sectional survey study | May 2020 | 1217 women | Convenience sample of middle-aged women, unweighted data | 1 | |
| Oldham *et al*. 2021 [33] | UK | Cross-sectional survey study | April to June 2020 | 2777 | Convenience sample of the general adult population, weighted data | 1A-1C | |
| Rathod *et al*. 2020 [34] | UK | Cross-sectional survey study | May to July 2020 | 3984 (subsample of non-healthcare professionals) | Convenience sample of the general adult population, unweighted data | 1 | |
| Robinson *et al*. 2020 [35] | UK | Cross-sectional survey study | April 2020 | 723 | Convenience sample of the general adult population, unweighted data | 1 | |
| Ingram *et al*. 2020 [36] | UK (Scotland) | Cross-sectional survey study | Spring 2020 | 399 | Convenience sample of the general adult population, weighted data | 1 | |
| **Southern European countries** | | | | | |  | |
| Đogaš *et al*. 2020 [37] | Croatia | Cross-sectional survey study | April to May 2020 | 3027 | Convenience sample of the general adult population, women being overrepresented, unweighted data | 2 | |
| Kolokotroni *et al*. 2021 [38] | Cyprus | Cross-sectional survey study | April to May 2020 | 745 | Convenience sample of the general adult population, women being overrepresented, unweighted data | 1A-1C, 2 | |
| Rantis *et al*. 2021 [39] | Greece | Cross-sectional survey study | April 2020 | 1043 (current drinker: 705) | Convenience sample of the general adult population, women being overrepresented, unweighted data | 1 | |
| Tsigkas *et al*. 2021 [40] | Greece (southwestern area) | Cross-sectional survey study | April 2020 | 1014 | General adult population sample (35+), weighted data | 1 | |
| Paltrinieri *et al*. 2021 [42] | Italy (Reggio Emilia) | Cross-sectional survey study | May to June 2020 | 1826 (current alcohol users: 956) | Convenience sample of the general adult population, women being overrepresented, unweighted data | 1A | |
| Rebelo *et al*. 2021 [43] | Portugal, Spain | Cross-sectional survey study focusing on wine consumption only | April to May 2020 | Portugal: 1940;  Spain: 2549 | Convenience sample of the general adult population, men being overrepresented, unweighted data; funded by industry | 1A, 2 | |
| Sande *et al*. 2021 [44] | Slovenia | Cross-sectional survey study | April to May 2020 | 680 (current alcohol users: 485) | Convenience sample of mostly young adult population, unweighted data | 1, 1C, 2 | |
| Observatorio Español de las Drogas y las Adicciones 2021 [45] | Spain | Cross-sectional survey study | November to December 2020 | 7886 (subsample of older adults: 894) | General adult population sample, weighted data | 1 | |
| Villanueva-Blasco *et al*. 2021 [46] | Spain | Cross-sectional survey study | April to May 2020 | 3779 (current alcohol users: 2345) | Convenience sample of the general adult population, weighted data | 2 | |
| García-Esquinas *et al*. 2020 [47] | Spain (Madrid; Madrid and Barcelona; Toledo) | Longitudinal survey study | April to June 2020 (compared to Madrid: 2019; Madrid and Barcelona: 2019-2020; Toledo: 2016-2017) | Madrid: 1323; Madrid and Barcelona: 464;  Toledo: 829 | General population sample of older adults, weighted data | 2 | |
| **Eastern European countries** | | | | | |  | |
| Makhashvili *et al*. 2020 [48] | Georgia | Cross-sectional survey study | May to June 2020 | 2088 | Convenience sample of the general adult population, unweighted data | 1 | |
| Chodkiewicz *et al*. 2020 [49] | Poland | Cross-sectional survey study | April 2020 | 443 | Convenience sample of the general adult population, unweighted data | 1 | |
| Dobrowolski & Włodarek 2021 [50] | Poland | Cross-sectional survey study | March to April 2020 | 184 | Convenience sample of the general adult population, women being overrepresented, unweighted data | 1 | |
| Kowalczuk & Gębski 2021 [51] | Poland | Cross-sectional survey study | March to May 2020 | 926 | Convenience sample of general adult population, women being overrepresented | 1 | |
| **Multiple countries covering more than one sub-European region** | | | | | |  | |
| Pišot *et al*. 2020 [52] | Europe (Bosnia and Herzegovina, Croatia, Greece, Kosovo, Italy, Serbia, Slovakia, Slovenia, Spain)^2^ | Cross-sectional survey study | April to May 2020 | 4108 (current alcohol users: 2773) | Convenience sample of the general adult population, unweighted data | 1 | |
| Janssen *et al*. 2021 [53] | Denmark, Germany, Slovenia | Cross-sectional survey study | April 2020 (Germany and Slovenia), April to May 2020 (Denmark) | Denmark: 1105; Germany: 973;  Slovenia: 602 | Convenience sample of the general adult population, unweighted data | 1 | |
| Manthey *et al*. 2021 [54] | Albania, Czechia, Denmark, Finland, France, Germany, Greece, Hungary, Iceland, Italy, Norway, Poland, Portugal, Russia, Slovakia, Slovenia, Spain, Sweden, UK | Cross-sectional survey study | April to July 2020 | 221 (Albania) to 15,762 (Norway); total sample size: 36,538 | Convenience sample of the general adult population, weighted data | 1A-1C | |
| Kilian *et al*. (*in press*) [55] | Belarus, Bulgaria, Croatia, Estonia, Georgia, Kosovo, Latvia, Lithuania, Moldova, Montenegro, Romania, Russia, Turkey, Ukraine | Cross-sectional survey study | August 2020 to January 2021 | 347 (Estonia) to 1988 (Latvia) | Convenience sample of the general adult population, weighted data | 1A-1C | |
| EIT Food Consumer Task Force 2021 [56] | Europe (Finland, France, Germany, Greece, Italy, Poland, Romania, Spain, Sweden, UK)^3^ | Cross-sectional survey study | September 2020 | 5000 | General adult population sample, weighted data | 1 | |

Note. ^1^ Number of current alcohol users indicated were assessed; ^2^ Type of change measure: 1 = proportional change in overall alcohol use, 1A = proportional change in drinking frequency, 1B = proportional change in drinking quantity, 1C = proportional change in heavy episodic drinking, 2 = mean change in prevalence of alcohol use; ^3^ only one estimate for all countries. UK, United Kingdom.

**Table S4.** Risk of bias (ROB) assessment.

| **Study** | **ROB** | **Critical ROB item** |
| --- | --- | --- |
| Alpers *et al*. 2021 | Serious | No information on missing values |
| Barrio *et al*. 2021 | Low |  |
| Belova *et al*. 2021 | Serious | No information on missing values, clinical sample |
| Benschop *et al*. 2021 | Serious | Non-probabilistic and unweighted sample |
| Blithikioti *et al*. 2021 | Low |  |
| Blom *et al*. 2021 | Serious | No information on missing values |
| Bramness *et al*. 2021 | Low |  |
| Carbia *et al*. 2021 | Serious | Non-probabilistic and unweighted sample |
| Central Statistics Office Ireland 2020 | Serious | No information on missing values |
| Chodkiewcz *et al*. 2021 | Serious | No information on missing values, selected sample (non-probabilistic and unweighted sample) |
| Cicero *et al*. 2020 | Serious | >20% missing values |
| Clay *et al*. 2021 | Serious | No information on missing values |
| Constant *et al*. 2020 | Serious | No information on missing values |
| Daly *et al*. 2021 | Serious | >20% missing values |
| Dobrowolski & Włodarek 2021 | Serious | Non-probabilistic and unweighted sample |
| Dogas *et al*. 2020 | Serious | No information on missing values |
| Drieskens *et al*. 2021 | Serious | No information on missing values |
| EIT Food Consumer Task Force 2021 | Serious | No information on missing values |
| Flycather Internet Research 2020a | Serious | No information on missing values |
| Flycather Internet Research 2020b | Serious | Non-probabilistic and unweighted sample, no information on missing values reported or >20% missing values |
| García-Esquinas *et al*. 2021 | Low |  |
| Garnett *et al*. 2021 | Moderate |  |
| Giacalone *et al*. 2020 | Serious | Non-probabilistic, unweighted sample |
| Guignard *et al*. 2021 | Low |  |
| Hakansson *et al*. 2020 | Serious | No information on missing values, selected sample (non-probabilistic and unweighted sample) |
| Ingram *et al*. 2020 | Serious | No information on missing values, selected sample (non-probabilistic and unweighted sample) |
| Jackson *et al*. 2021 | Low |  |
| Janssen *et al*. 2021 | Moderate |  |
| Kilian *et al*. (*in press*) | Moderate |  |
| Klosterhalfen *et al*. 2021 | Moderate |  |
| Kolokotroni *et al*. 2021 | Serious | No information on missing values |
| Koopmann *et al*. 2021 | Moderate |  |
| Kowalczuk & Gębski 2021 | Serious | No information on missing values, selected sample (non-probabilistic and unweighted sample) |
| Kriaucioniene *et al*. | Serious | No information on missing values, selected sample (non-probabilistic and unweighted sample) |
| Laghi *et al*. 2021 | Moderate |  |
| López-Bueno *et al*. 2020 | Low |  |
| Mäkelä *et al*. 2021 | Serious | No information on sampling and missing values |
| Makhashvili *et al*. 2020 | Serious | No information on missing values |
| Manthey *et al*. 2021 | Low |  |
| Marty *et al*. 2021 | Serious | 30% missings |
| Merlo *et al*. 2021 | Serious | Non-probabilistic and unweighted sample, no information on missing values reported or >20% missing values |
| Miller *et al*. 2021 | Serious | No information on missing values reported or >20% missing values |
| Norstat 2021 | Serious | No information on missing values |
| Observatorio Español de las Drogas y las Adicciones 2021 | Serious | 40% missings |
| Oksanen *et al*. 2021 | Low |  |
| Oldham *et al*. 2021 | Low |  |
| Opinion 2020 | Serious | No information on missing values |
| Pabst *et al*. 2021a | Serious | More than >20% missing values |
| Pabst *et al*. 2021b | Low |  |
| Paltrinieri al. 2021 | Moderate |  |
| Pisot *et al*. 2020 | Serious | Non-probalistic, unweighted sample, >20% missing values |
| Profeta *et al*. 2021 | Serious | Non-probabilistic and unweighted sample |
| Rantis *et al*. 2021 | Serious | Non-probabilistic and unweighted sample, no information on missing values reported or >20% missing values |
| Rao *et al*. 2021 | Moderate |  |
| Rathod *et al*. 2020 | Serious | Non-probalistic, unweighted sample, >20% missing values |
| Rebelo *et al*. 2021 | Serious | Non-probabilistic and unweighted sample, no information on missing values reported or >20% missing values, subjective assessment of changes in alcohol use, and other systematic error in outcome measurement |
| Reynolds *et al*. 2021 | Low |  |
| Robinson *et al*. 2020 | Serious | Non-probalistic, unweighted sample, >20% missing values |
| Rolland *et al*. 2020 | Serious | >20% missing values |
| Rossinot *et al*. 2020 | Low |  |
| Sande *et al*. 2021 | Serious | Non-probabilistic and unweighted sample |
| Schecke *et al*. 2021 | Moderate |  |
| Schiestl 2020 | Serious | No information on sampling and missing values |
| Sidor *et al*. 2020 | Serious | Non-probalistic, unweighted sample |
| Skotnicka *et al*. 2021 | Serious | Non-probabilistic and unweighted sample, no information on missing values reported or >20% missing values |
| Strizek *et al*. 2021 | Serious | No information on missing values reported or >20% missing values |
| Studer *et al*. 2021 | Low |  |
| Tsigkas *et al*. 2021 | Moderate |  |
| Vanderbruggen *et al*. 2020 | Serious | Non-probalistic, unweighted sample |
| Villanueva 2021 | Low |  |
| Villanueva-Blasco *et al*. 2021 | Moderate |  |
| Winkler *et al*. 2021 | Serious | No information on missing values reported or >20% missing values |

**Table S5.** Results for random-effects meta-regression analyses on the pooled change in overall alcohol use (total sample; 44 studies).

| **Moderator** | **Estimate** | **95% CI** | ***P*-value** | **I^2^** |
| --- | --- | --- | --- | --- |
| *Age distribution (ref.: general population sample)* |  |  |  |  |
| Sample of younger adults | -0.025 | -0.133, 0.083 | 0.650 | 99.4% |
| Sample of older adults | 0.032 | -0.111, 0.176 | 0.653 |  |
| Consideration of weights (ref.: no): yes | -0.014 | -0.090, 0.063 | 0.719 | 99.4% |
| *Study period (ref.: March to June 2020)* |  |  |  |  |
| July to September 2020 | -0.063 | -0.171, 0.046 | 0.252 | 99.4% |
| October 2020 or later | -0.119 | -0.244, 0.006 | 0.062 |  |
| *Sub-European region (ref.: central and eastern European region), n = 42^1^* |  |  |  |  |
| Northern European region | 0.003 | -0.134, 0.140 | 0.970 | 99.3% |
| Southern European region | -0.160 | -0.329, 0.009 | 0.062 |  |
| Western European region | 0.008 | -0.133, 0.149 | 0.911 |  |

Note: ^1^Studies including estimates for multiple countries were excluded. CI, confidence interval.

**Table S6.** Results for random-effects meta-regression analyses on the pooled change in overall alcohol use (women; 15 studies).

| **Moderator** | **Estimate** | **95% CI** | ***P*-value** | **I^2^** |
| --- | --- | --- | --- | --- |
| *Age distribution (ref.: general population sample)* |  |  |  |  |
| Sample of younger adults | -0.108 | -0.372, 0.157 | 0.392 | 99.1% |
| Sample of older adults | 0.007 | -0.257, 0.271 | 0.952 |  |
| Consideration of weights (ref.: no): yes | -0.061 | -0.236, 0.113 | 0.463 | 99.2% |
| *Study period (ref.: March to June 2020)* |  |  |  |  |
| July to September 2020 | -0.144 | -0.397, 0.109 | 0.239 | 99.3% |
| October 2020 or later | -0.110 | -0.325, 0.104 | 0.284 |  |
| *Sub-European region (ref.: central and eastern European region)* |  |  |  |  |
| Northern European region | 0.224 | -0.117, 0.565 | 0.176 | 99.1% |
| Southern European region | 0.058 | -0.333, 0.448 | 0.751 |  |
| Western European region | 0.230 | -0.119, 0.579 | 0.175 |  |

CI, confidence interval.

**Table S7.** Results for random-effects meta-regression analyses on the pooled change in overall alcohol use (men; 13 studies).

| **Moderator** | **Estimate** | **95% CI** | ***P*-value** | **I^2^** |
| --- | --- | --- | --- | --- |
| *Age distribution (ref.: general population sample)* |  |  |  |  |
| Sample of younger adults | -0.062 | -0.318, 0.193 | 0.598 | 98.5% |
| Sample of older adults | -0.020 | -0.360, 0.320 | 0.897 |  |
| Consideration of weights (ref.: no): yes | -0.106 | -0.264, 0.052 | 0.166 | 98.5% |
| *Study period (ref.: March to June 2020)* |  |  |  |  |
| July to September 2020 | -0.009 | -0.227, 0.209 | 0.930 | 98.4% |
| October 2020 or later | -0.192 | -0.409, 0.026 | 0.078 |  |
| *Sub-European region (ref.: central and eastern European region)* |  |  |  |  |
| Northern European region | 0.218 | -0.083, 0.519 | 0.136 | 98.2% |
| Southern European region | 0.043 | -0.296, 0.382 | 0.779 |  |
| Western European region | 0.243 | -0.068, 0.554 | 0.111 |  |

CI, confidence interval.

**Table S8.** Results for random-effects meta-regression analyses on the pooled change in drinking frequency (7 studies).

| **Moderator** | **Estimate** | **95% CI** | ***P*-value** | **I^2^** |
| --- | --- | --- | --- | --- |
| *Age distribution (ref.: general population sample)* |  |  |  |  |
| Sample of younger adults | -0.057 | -0.403, 0.290 | 0.743 | 98.4% |
| Sample of older adults^1^ | . | . | . |  |
| Consideration of weights (ref.: no): yes | -0.075 | -0.234, 0.085 | 0.349 | 98.3% |
| *Study period (ref.: March to June 2020)* |  |  |  |  |
| July to September 2020 | -0.079 | -0.311, 0.154 | 0.497 | 98.2% |
| October 2020 or later | -0.136 | -0.241, -0.032 | 0.012 |  |
| *Sub-European region (ref.: central and eastern European region)* |  |  |  |  |
| Northern European region | 0.066 | -0.065, 0.197 | 0.315 | 98.2% |
| Southern European region | -0.041 | -0.168, 0.086 | 0.516 |  |
| Western European region | 0.175 | -0.075, 0.425 | 0.165 |  |

Note: ^1^There was no study including older adults only. CI, confidence interval.

**Table S9.** Results for random-effects meta-regression analyses on the pooled change in the amount of alcohol consumed per drinking occasion (6 studies).

| **Moderator** | **Estimate** | **95% CI** | ***P*-value** | **I^2^** |
| --- | --- | --- | --- | --- |
| *Age distribution (ref.: general population sample)* |  |  |  |  |
| Sample of younger adults^1^ | . | . | . | 97.6% |
| Sample of older adults | 0.023 | -0.227, 0.273 | 0.853 |  |
| Consideration of weights (ref.: no): yes | 0.039 | -0.109, 0.187 | 0.597 | 97.5% |
| *Study period (ref.: March to June 2020)* |  |  |  |  |
| July to September 2020 | 0.013 | -0.166, 0.191 | 0.888 | 97.3% |
| October 2020 or later | -0.070 | -0.151, 0.01 | 0.085 |  |
| *Sub-European region (ref.: central and eastern European region)* |  |  |  |  |
| Northern European region | 0.064 | -0.032, 0.161 | 0.185 | 97.2% |
| Southern European region | 0.007 | -0.090, 0.104 | 0.889 |  |
| Western European region | 0.156 | -0.029, 0.341 | 0.095 |  |

Note: ^1^There was no study including younger adults only. CI, confidence interval.

**Table S10.** Results for random-effects meta-regression analyses on the pooled change in the frequency of heavy episodic drinking (6 studies).

| **Moderator** | **Estimate** | **95% CI** | ***P-value*** | **I^2^** |
| --- | --- | --- | --- | --- |
| *Age distribution (ref.: general population sample)* |  |  |  |  |
| Sample of younger adults^1^ | . | . | . |  |
| Sample of older adults^1^ | . | . | . |  |
| Consideration of weights (ref.: no): yes | 0.135 | -0.047, 0.317 | 0.141 | 98.3% |
| *Study period (ref.: March to June 2020)* |  |  |  |  |
| July to September 2020 | -0.025 | -0.221, 0.171 | 0.798 | 98.2% |
| October 2020 or later | 0.006 | -0.082, 0.094 | 0.895 |  |
| *Sub-European region (ref.: central and eastern European region)* |  |  |  |  |
| Northern European region | 0.010 | -0.098, 0.117 | 0.857 | 98.1% |
| Southern European region | 0.021 | -0.087, 0.128 | 0.700 |  |
| Western European region | 0.049 | -0.156, 0.254 | 0.632 |  |

Note: ^1^There was no study including younger or older adults only. CI, confidence interval.

**Table S11.** Results for random-effects meta-regression analyses on the pooled change in the prevalence of alcohol users during versus before the pandemic (7 studies).

| **Moderator** | **Estimate** | **95% CI** | ***P-value*** | **I^2^** |
| --- | --- | --- | --- | --- |
| *Age distribution (ref.: general population sample)* |  |  |  |  |
| Sample of younger adults | 0.025 | -0.079, 0.129 | 0.592 | 72.2% |
| Sample of older adults | 0.091 | 0.006, 0.175 | 0.039 |  |
| Consideration of weights (ref.: no): yes | 0.037 | -0.054, 0.129 | 0.372 | 82.6% |
| *Study period (ref.: March to June 2020)* |  |  |  |  |
| July to September 2020 | 0.044 | -0.102, 0.189 | 0.500 | 78.4% |
| October 2020 or later | 0.057 | -0.055, 0.169 | 0.266 |  |
| *Sub-European region (ref.: central and eastern European region)* |  |  |  |  |
| Northern European region^1^ | . | . | . | 83.1% |
| Southern European region^1^ | . | . | . |  |
| Western European region | 0.029 | -0.116, 0.174 | 0.656 |  |

Note: ^1^There was no study located in the Northern and Southern European region. CI, confidence interval.

**Table S12.** Key characteristics of studies included in the narrative summary of alcohol use changes among people with alcohol use disorder.

| **Study** | **Country** | **Study design** | **Study period** | **Sample size** | **Sample characteristics** |
| --- | --- | --- | --- | --- | --- |
| Pabst *et al*. 2021 [57] | Belgium | Cross-sectional survey study | April and May 2020 | 7718 (AUD: 299; moderate drinkers: 299) | Convenience sample of general adult population (AUD and matched moderate drinker sub-samples) |
| Winkler *et al*. 2021 [58] | Czechia | Repeated cross-sectional survey study | May and November 2020 (compared to November 2017) | May 2020: 3021; November 2020: 3000 November 2017: 3306 | General adult population sample, weighted data |
| Sidor & Rzymski 2020 [59] | Poland | Cross-sectional survey study | March to May 2020 | 1097 (including 14 self-reported alcohol dependent drinkers) | Convenience sample of the general adult population, including a small group of people with self-reported 'alcohol addiction' |
| Belova *et al*. 2021 [60] | Russia | Assessment of patient records | March to May 2020 (compared to the same period in 2019) | 786 patients in 2019 and 774 patients in 2020 | Sample of adult patients who were treated in the Department of Acute Toxicosis and Somatopsychiatric Disorders of the N. V. Sklifosovsky Research, Institute for Emergency Medicine |
| Barrio *et al*. 2021 [61] | Spain (Barcelona) | Retrospective cohort study | January to June 2020 | 362 | Adult patients attending Addictions Unit outpatient service at specialist hospital |
| Blithikioti *et al*. 2021 [62] | Spain (Barcelona) | Cross-sectional survey study | June to July 2020 | 303 (46% seeking help for alcohol use disorder) | SUD patients attending Addiction Unit outpatient service of University Barcelona Clínic Hospital |
| Jackson *et al*. 2021 [43] | UK (England) | Repeated cross-sectional survey study | April to July 2020 (compared to August 2019 to February 2020, and August 2018 to February 2019 and April to July 2019) | 35,980 adults from the general population, weighted data | Significant increase in the prevalence of high-risk drinking by +39.5% during COVID-19 lockdown (compared to -7.8% in comparator year 2018/2019), with greater increase in women (+55.4%) compared to men (+30.7%).  Significant increase in alcohol reduction attempts (+75.5%) |
| Rao *et al*. 2021 [63] | UK (South London) | Assessment of patient records attending mental health service | October 2019 to August 2020 | 366 (pre-pandemic: 185, during pandemic: 181) | Clinical sample of older adults ("baby boomer" generation) attending the mental health services and who had consumed alcohol in the past year |

AUD, alcohol use disorder; SUD, substance use disorder; UK, United Kingdom.

Figure S1. Sensitivity analysis: random-effects meta-analysis for changes in alcohol use (n = 21); reports based on convenience samples were excluded. Outcome measure was the difference in the proportion of respondents reporting increases minus decreases in alcohol use.

Figure S2. Random-effects meta-analysis for changes in alcohol use among women. Outcome measure was the difference in the proportion of respondents reporting increases minus decreases in alcohol use.

Figure S3. Random-effects meta-analysis for changes in alcohol use among men. Outcome measure was the difference in the proportion of respondents reporting increases minus decreases in alcohol use.

Figure S4. Funnel plot for random-effects meta-analysis for changes in alcohol use for the total sample.

Figure S5. Funnel plot for sensitivity analysis.

Figure S6. Funnel plot for random-effects meta-analysis for changes in alcohol use for women.

Figure S7. Funnel plot for random-effects meta-analysis for changes in alcohol use for men.

Figure S8. Random-effects meta-analysis for changes in the drinking frequency. Outcome measure was the difference in the proportion of respondents reporting increases minus decreases in their drinking frequency.

Figure S9. Random-effects meta-analysis for changes in the drinking quantity. Outcome measure was the difference in the proportion of respondents reporting increases minus decreases in their drinking quantity.

Figure S10. Random-effects meta-analysis for changes in the frequency of heavy episodic drinking. Outcome measure was the difference in the proportion of respondents reporting increases minus decreases in their frequency of heavy episodic drinking.

Figure S11. Funnel plot for random-effects meta-analysis for changes in drinking frequency.

Figure S12. Funnel plot for random-effects meta-analysis for changes in drinking quantity.

Figure S13. Funnel plot for random-effects meta-analysis for changes in frequency of heavy episodic drinking.

Figure S14. Random-effects meta-analysis for changes in the prevalence of alcohol use. Outcome measure was the difference in the proportion of respondents reporting alcohol use during minus before the pandemic period.

Figure S15. Funnel plot for random-effects meta-analysis for changes in prevalence of alcohol use.

# References

[1] Schiestl DW. Wie entwickelt sich der Konsum von Alkohol und Tabak in der Krise? Ein Update. Corona Blog, 2020. Available at: https://viecer.univie.ac.at/corona-blog/corona-blog-beitraege/blog27/.

[2] Strizek J, Busch M, Puhm A, *et al*. Repräsentativerhebung zu Konsum‐ und Verhaltensweisen mit Suchtpotenzial. Wien, Österreich: Gesundheit Österreich GmbH, 2021.

[3] Drieskens S, Berger N, Vandevijvere S, *et al*. Short-term impact of the COVID-19 confinement measures on health behaviours and weight gain among adults in Belgium. Arch Public Health 2021;79:22.

[4] Pabst A, Bollen Z, Creupelandt C, *et al*. Alcohol consumption changes during the first COVID-19 lockdown: an online population survey in a convenience sample of French-speaking Belgian residents. Psychiatry Research 2021;300:113938.

[5] Vanderbruggen N, Matthys F, Van Laere S, *et al*. Self-reported alcohol, tobacco, and cannabis use during COVID-19 lockdown measures: Results from a web-based survey. Eur Addict Res 2020;26:309–15.

[6] Guignard R, Andler R, Quatremère G, *et al*. Changes in smoking and alcohol consumption during COVID-19-related lockdown: a cross-sectional study in France. Eur J Public Health 2021;31:1076-83.

[7] Rolland B, Haesebaert F, Zante E, *et al*. Global Changes and Factors of Increase in Caloric/Salty Food Intake, Screen Use, and Substance Use During the Early COVID-19 Containment Phase in the General Population in France: Survey Study. JMIR Public Health Surveill 2020;6:e19630.

[8] Rossinot H, Fantin R, Venne J. Behavioral changes during COVID-19 confinement in France: A web-based study. Int J Environ Res Public Health 2020;17:8444.

[9] Constant A, Conserve DF, Gallopel-Morvan K, *et al*. Socio-Cognitive Factors Associated With Lifestyle Changes in Response to the COVID-19 Epidemic in the General Population: Results From a Cross-Sectional Study in France. Front Psychol 2020;11:579460.

[10] Klosterhalfen S, Kastaun S, Kotz D. Did self‐reported tobacco smoking, alcohol consumption, and physical activity change during the COVID-19 restrictions in Germany in spring 2020? Findings from a population survey (the DEBRA study). Preprint, In Review. Epub ahead of print 12 May 2021. DOI: 10.21203/rs.3.rs-479652/v1.

[11] Koopmann A, Georgiadou E, Reinhard I, *et al*. The Effects of the Lockdown during the COVID-19 Pandemic on Alcohol and Tobacco Consumption Behavior in Germany. Eur Addict Res 2021;27:242–56.

[12] Profeta A, Siddiqui SA, Smetana S, *et al*. The impact of Corona pandemic on consumer’s food consumption: Vulnerability of households with children and income losses and change in sustainable consumption behavior. J Consum Prot Food Saf. 2021 [Epub ahead of print].

[13] Schecke H, Fink M, Bäuerle A, *et al*. Changes in Substance Use and Mental Health Burden among Women during the Second Wave of COVID-19 in Germany. Int J Environ Res Public Health 2021;18:9728.

[14] Benschop A, van Bakkum F, Noijen J. Changing Patterns of Substance Use During the Coronavirus Pandemic: Self-Reported Use of Tobacco, Alcohol, Cannabis, and Other Drugs. Front Psychiatry 2021;12:633551.

[15] Flycatcher Internet Research. Eetgedrag en corona. Maastricht: Flycatcher Internet Research, 2020. Available at: https://www.voedingscentrum.nl/Assets/Uploads/voedingscentrum/Documents/Professionals/Pers/Persmappen/Rapportage%20-%20Eetgedrag%20en%20corona%20(mei%202020).pdf.

[16] Merlo A, Hendriksen PA, Severeijns NR, *et al*. Alcohol Consumption Patterns during COVID-19 Lockdown and Their Relationship with Perceived Immune Fitness and Reported COVID-19 Symptoms. Healthcare 2021;9:1039.

[17] Giacalone D, Frøst MB, Rodríguez-Pérez C. Reported Changes in Dietary Habits During the COVID-19 Lockdown in the Danish Population: The Danish COVIDiet Study. Front Nutr 2020;7:592112.

[18] Mäkelä P, Ylöstalo T, Warpenius K, *et al*. Koronaepidemian vaikutukset suomalaisten alkoholinkulutukseen – kokonaisarvio kevään ja kesän 2020 muutoksista [Impacts of the COVID-19 pandemic on alcohol consumption in Finland – an overview of changes in spring and summer 2020]. Helsinki: Finnish Institute for Health and Welfare., 2020.

[19] Oksanen A, Savolainen I, Savela N, *et al*. Psychological stressors predicting increased drinking during the COVID-19 crisis: A longitudinal national survey study of workers in Finland. Alcohol Alcohol 2021;56:299–306.

[20] Carbia C, García-Cabrerizo R, Cryan JF, *et al*. Associations between mental health, alcohol consumption and drinking motives during COVID-19 second lockdown in Ireland. Alcohol Alcohol 2021 [Epub ahead of print].

[21] Central Statistics Office. Social Impact of COVID-19 Survey. Cork, Ireland: Central Statistics Office, 2020. Available at: https://www.cso.ie/en/csolatestnews/presspages/2020/socialimpactofcovid-19surveynovember2020well-beingandlifestyleunderlevel5restrictions/.

[22] Reynolds CME, Purdy J, Rodriguez L, *et al*. Factors associated with changes in consumption among smokers and alcohol drinkers during the COVID-19 ‘lockdown’ period. Eur J Public Health 2021;31:1084–9.

[23] Kriaucioniene V, Bagdonaviciene L, Rodríguez-Pérez C, *et al*. Associations between changes in health behaviours and body weight during the COVID-19 quarantine in Lithuania: The Lithuanian COVIDiet Study. Nutrients 2020; 2:3119.

[24] Bramness JG, Bye EK, Moan IS, Rossow I. Alcohol use during the COVID-19 pandemic: Self-reported changes and motives for change. Eur Addict Res 2021;27:257-62.

[25] Opinion. Befolkningsundersøkelse om alkohol i koronaepidemien. Oslo, Norway: Opinion AS, 2020.

[26] Alpers SE, Skogen JC, Mæland S, *et al*. Alcohol consumption during a pandemic lockdown period and change in alcohol consumption related to worries and pandemic measures. Int J Environ Res Public Health 2021;18:1220.

[27] Blom V, Lönn A, Ekblom B, *et al*. Lifestyle habits and mental health in light of the two COVID-19 pandemic waves in Sweden, 2020. Int J Environ Res Public Health 2021;18:3313.

[28] Håkansson A. Changes in gambling behavior during the COVID-19 pandemic—A web survey study in Sweden. Int J Environ Res Public Health 2020;17:4013.

[29] Norstat. Undersökning i befolkningen om alkoholvanor 2020. 2020.

[30] Clay JM, Stafford LD, Parker MO. Associations between self-reported inhibitory control, stress, and alcohol (mis)use during the first wave of the COVID-19 pandemic in the UK: a National cross-sectional study utilising data from four birth cohorts. Int J Ment Health Addiction 2021 [Epub ahead of print].

[31] Garnett C, Jackson S, Oldham M, *et al*. Factors associated with drinking behaviour during COVID-19 social distancing and lockdown among adults in the UK. Drug Alcohol Depend 2021;219:108461.

[32] Miller ER, Olver IN, Wilson CJ, *et al*. COVID-19, Alcohol Consumption and Stockpiling Practises in Midlife Women: Repeat Surveys During Lockdown in Australia and the United Kingdom. Front Public Health 2021;9:642950.

[33] Oldham M, Garnett C, Brown J, *et al*. Characterising the patterns of and factors associated with increased alcohol consumption since COVID ‐19 in a UK sample. Drug Alcohol Rev 2021;40:890–9.

[34] Rathod S, Pallikadavath S, Young AH, *et al*. Psychological impact of COVID-19 pandemic: Protocol and results of first three weeks from an international cross-section survey - focus on health professionals. J Affect Disord Rep 2020;1:100005.

[35] Robinson E, Gillespie S, Jones A. Weight‐related lifestyle behaviours and the COVID‐19 crisis: An online survey study of UK adults during social lockdown. Obes Sci Pract 2020;6:735–40.

[36] Ingram J, Maciejewski G, Hand CJ. Changes in diet, sleep, and physical activity are associated with differences in negative mood during COVID-19 lockdown. Front Psychol 2020;11:588604.

[37] Đogaš Z, Lušić Kalcina L, Pavlinac Dodig I, *et al*. The effect of COVID-19 lockdown on lifestyle and mood in Croatian general population: a cross-sectional study. Croat Med J 2020;61:309–18.

[38] Kolokotroni O, Mosquera MC, Quattrocchi A, Heraclides A, Demetriou C, Philippou E. Lifestyle habits of adults during the COVID-19 pandemic lockdown in Cyprus: Evidence from a cross-sectional study. BMC Public Health 2021;21:786.

[39] Rantis K, Panagiotidis P, Parlapani E, Holeva V, Tsapakis EM, Diakogiannis I. Substance use during the COVID-19 pandemic in Greece. J Subst Use 2021 [Epub ahead of print].

[40] Tsigkas G, Koufou E-E, Katsanos K, *et al*. Potential relationship between lifestyle changes and incidence of hospital admissions for acute coronary syndrome during the COVID-19 lockdown. Front Cardiovasc Med 2021;8:604374.

[41] Cicero A, Fogacci F, Giovannini M, *et al*. COVID-19-related quarantine effect on dietary habits in a Northern Italian rural population: Data from the Brisighella Heart Study. Nutrients 2021;13:309.

[42] Paltrinieri S, Bressi B, Costi S, *et al*. Beyond lockdown: The potential side effects of the SARS-CoV-2 pandemic on public health. Nutrients 2021;13:1600.

[43] Rebelo J, Compés R, Faria S, *et al*. Covid-19 lockdown and wine consumption frequency in Portugal and Spain. Span J Agric Res 2021;19:e0105R.

[44] Sande M, Šabić S, Paš M, *et al*. How has the COVID-19 Epidemic Changed Drug Use and the Drug Market in Slovenia? DI 2021;30:313–32.

[45] Observatorio Español de las Drogas y las Adicciones. Encuesta OEDA-COVID: Impacto de la pandemia por Covid-19 durante el año 2020 en el patrón de consumo de sustancias psicoactivas y otros comportamientos con potencial adictivo. Madrid, Spain: Ministerio de Sanidad. Delegación del Gobierno para el Plan Nacional sobre Drogas, 2021.

[46] Villanueva-Blasco VJ, Villanueva Silvestre V, Isorna M, *et al*. Changes in Alcohol Consumption Pattern Based on Gender during COVID-19 Confinement in Spain. Int J Environ Res Public Health 2021;18:8028.

[47] García-Esquinas E, Ortolá R, Gine-Vázquez I, *et al*. Changes in Health Behaviors, Mental and Physical Health among Older Adults under Severe Lockdown Restrictions during the COVID-19 Pandemic in Spain. Int J Environ Res Public Health 2021;18:7067.

[48] Makhashvili N, Javakhishvili JD, Sturua L, *et al*. The influence of concern about COVID-19 on mental health in the Republic of Georgia: a cross-sectional study. Global Health 2020;16:111.

[49] Chodkiewicz J, Talarowska M, Miniszewska J, *et al*. Alcohol Consumption Reported during the COVID-19 Pandemic: The Initial Stage. Int J Environ Res Public Health 2020;17:4677.

[50] Dobrowolski H, Włodarek D. Body Mass, Physical Activity and Eating Habits Changes during the First COVID-19 Pandemic Lockdown in Poland. Int J Environ Res Public Health 2021;18:5682.

[51] Kowalczuk I, Gębski J. Impact of fear of contracting COVID-19 and complying with the rules of isolation on nutritional behaviors of Polish adults. Int J Environ Res Public Health 2021;18:1631.

[52] Pišot S, Milovanović I, Šimunič B, *et al*. Maintaining everyday life praxis in the time of COVID-19 pandemic measures (ELP-COVID-19 survey). Eur J Public Health 2020;30:1181–6.

[53] Janssen M, Chang BPI, Hristov H, *et al*. Changes in food consumption during the COVID-19 pandemic: Analysis of consumer survey data from the first lockdown period in Denmark, Germany, and Slovenia. Front Nutr 2021;8:635859.

[54] Manthey J, Kilian C, Carr S, *et al*. Use of alcohol, tobacco, cannabis, and other substances during the first wave of the SARS-CoV-2 pandemic in Europe: A survey on 36,000 European substance users. Subst Abuse Treat Prev Policy 2021;16:36.

[55] Kilian C, Neufeld M, Manthey J, *et al*. Self-reported changes in alcohol and tobacco use during COVID-19: findings from the eastern part of WHO European Region. Eur J Public Health [In press].

[56] EIT Food Consumer Task Force. COVID-19 impact on consumer food behaviours in Europe. European Institute of Innovation and Technology (EIT), 2021. Available at: eitfood.eu/projects/how-the-corona-crisis-affectsconsumer- behaviour-and-consumer-demand-forfood- products-and-services.

[57] Pabst A, Bollen Z, Creupelandt C, Fontesse S, Maurage P. Alcohol consumption changes following COVID-19 lockdown among French-speaking Belgian individuals at risk for alcohol use disorder. Prog Neuropsychopharmacol Biol Psychiatry 2021;119:110282

[58] Winkler P, Mohrova Z, Mlada K, *et al*. Prevalence of current mental disorders before and during the second wave of COVID-19 pandemic: An analysis of repeated nationwide cross-sectional surveys. J Psychiatr Res 2021;139:167–71.

[59] Sidor A, Rzymski P. Dietary choices and habits during COVID-19 lockdown: Experience from Poland. Nutrients 2020;12:1657.

[60] Belova MV, Ilyashenko KK, Simonova AY, Potskhveriya M, Trusov G. The structure of acute exotoxicosis during the first three months of the COVID-19 pandemic (according to the Acute Toxicosis Department of N.V. Sklifosovsky Research Institute for Emergency Medicine). Sklifosovsky J Emergy Medical Care 2021;10:27–32.

[61] Barrio P, Baldaquí N, Andreu M, *et al*. Abstinence among alcohol use disorder patients during the COVID‐19 pandemic: insights from Spain. Alcohol Clin Exp Res 2021;45:802-7.

[62] Blithikioti C, Nuño L, Paniello B, *et al*. Impact of COVID-19 lockdown on individuals under treatment for substance use disorders: Risk factors for adverse mental health outcomes. J Psychiatr Res 2021;139:47-53.

[63] Rao R, Mueller C, Broadbent M. Risky alcohol consumption in older people before and during the COVID-19 pandemic in the United Kingdom. J Subst Use 2021 [Epub ahead of print].
